# The Type III Secretion Translocation Pore Senses Host Cell Contact

**DOI:** 10.1371/journal.ppat.1005530

**Published:** 2016-03-29

**Authors:** Erin I. Armentrout, Arne Rietsch

**Affiliations:** Department of Molecular Biology and Microbiology, Case Western Reserve University, Cleveland, Ohio, United States of America; Osaka University, JAPAN

## Abstract

Type III secretion systems (T3SS) are nano-syringes used by a wide range of Gram-negative pathogens to promote infection by directly injecting effector proteins into targeted host cells. Translocation of effectors is triggered by host-cell contact and requires assembly of a pore in the host-cell plasma membrane, which consists of two translocator proteins. Our understanding of the translocation pore, how it is assembled in the host cell membrane and its precise role in effector translocation, is extremely limited. Here we use a genetic technique to identify protein-protein contacts between pore-forming translocator proteins, as well as the T3SS needle-tip, that are critical for translocon function. The data help establish the orientation of the translocator proteins in the host cell membrane. Analysis of translocon function in mutants that break these contacts demonstrates that an interaction between the pore-forming translocator PopD and the needle-tip is required for sensing host cell contact. Moreover, tethering PopD at a dimer interface also specifically prevents host-cell sensing, arguing that the translocation pore is actively involved in detecting host cell contact. The work presented here therefore establishes a signal transduction pathway for sensing host cell contact that is initiated by a conformational change in the translocation pore, and is subsequently transmitted to the base of the apparatus via a specific contact between the pore and the T3SS needle-tip.

## Author Summary

Type III secretion systems (T3SSs) are molecular syringes used by a wide variety of Gram-negative pathogens to directly deliver proteins (effectors) into host cells, allowing the bacteria to cause disease. Injection of proteins is triggered by host-cell contact, but how the machinery to deliver effectors is assembled (the translocon), or indeed, how cell contact is perceived, is unclear. Here we identify protein-protein contacts that are critical for translocon function. Our analysis sheds light on the organization of the translocon, and reveals that host cell contact is perceived by a change in the structure of the translocation pore. This signal is then transmitted to the tip of the T3SS needle, and down to the base of the apparatus.

## Introduction

Type III secretion systems (T3SSs) are molecular syringes employed by a wide range of Gram-negative pathogens to directly inject effector proteins into targeted host cells, and thereby promote disease [1, 2]. A hallmark of these virulence-associated T3SSs is that effector secretion is triggered by host cell contact, and that injection of effector proteins into the cell is vectorial [3]. Only T3SS that engage the host cell trigger effector secretion, and effectors are injected into the host cell, but not into the surrounding milieu.

The structure that facilitates this directed injection of effector proteins is the translocon [4–8]. Upon cell contact, the T3SS needle is brought into close proximity of the host cell plasma membrane. The specialized tip structure at the distal end of the T3SS needle facilitates the insertion of the pore forming translocator proteins into the targeted host cell membrane [9–11]. Animal pathogens produce two hydrophobic translocators that assemble to form the translocation pore [7, 12, 13]. The pore likely docks to the needle tip, forming the translocon [14, 15], through which effectors are subsequently injected into the host cell. Atomic force imaging of pores assembled by enteropathogenic *E*. *coli* into red blood cell membranes suggests that the pores have a 6–8 fold symmetry [16]. Triggering of effector secretion depends on the translocon, and does not occur if the translocon is not assembled [17].

Our understanding of the translocon is limited. While pore forming translocator proteins can insert into membranes *in vitro* [18–20] the organization of the translocation pore, including the stoichiometry of the pore forming translocators, and in some instances, their orientation in the membrane are unknown. Moreover, the behavior of translocator proteins *in vitro* does not perfectly mimic observations made in the context of bacterial infection. For example, while the *Pseudomonas aeruginosa* pore-forming translocator proteins, PopB and PopD, can form pores individually in lipid vesicles *in vitro* [18], pore formation by translocator proteins delivered by the bacterium, requires both PopB and PopD [9]. Similarly, while PcrV facilitates translocator insertion [9, 10], it does not interact with PopB or PopD *in vitro* [18, 21]. In a similar vein, potential triggers for effector secretion have been proposed for a number of T3SS [22, 23], however, it is not known what component of the T3SS senses host cell contact to initiate injection of effector proteins.

Here we use a genetic approach to map interactions between translocator proteins that are critical for the delivery of effectors into host cells. The approach is based on the fact that translocator proteins between closely related T3SS are often unable to cross-complement [24–26]. We exploited the incompatibility of the translocator proteins of *P*. *aeruginosa* and *Yersinia pseudotuberculosis*, to map critical interactions by generating hybrid proteins that restore translocon function. In this manner we mapped two contacts, one between the needle tip protein PcrV and the pore-forming translocator protein PopD, as well as a second region of contact between PopB and PopD. We also determined that both PopB and PopD form dimers. Importantly, we demonstrate that tethering the PopD dimer by forming a disulfide bond specifically interferes with triggering of effector secretion. Similarly, breaking the PopD-PcrV contact prevents transmission of the host-cell contact signal to commence effector secretion. Our data support a model in which the translocation pore is actively involved in sensing host cell contact, a signal that is transmitted to the base of the apparatus via contacts between the pore-forming translocator proteins and the needle tip.

## Results

### Translocator-interactions that are critical for T3SS function

**Fig 1:**
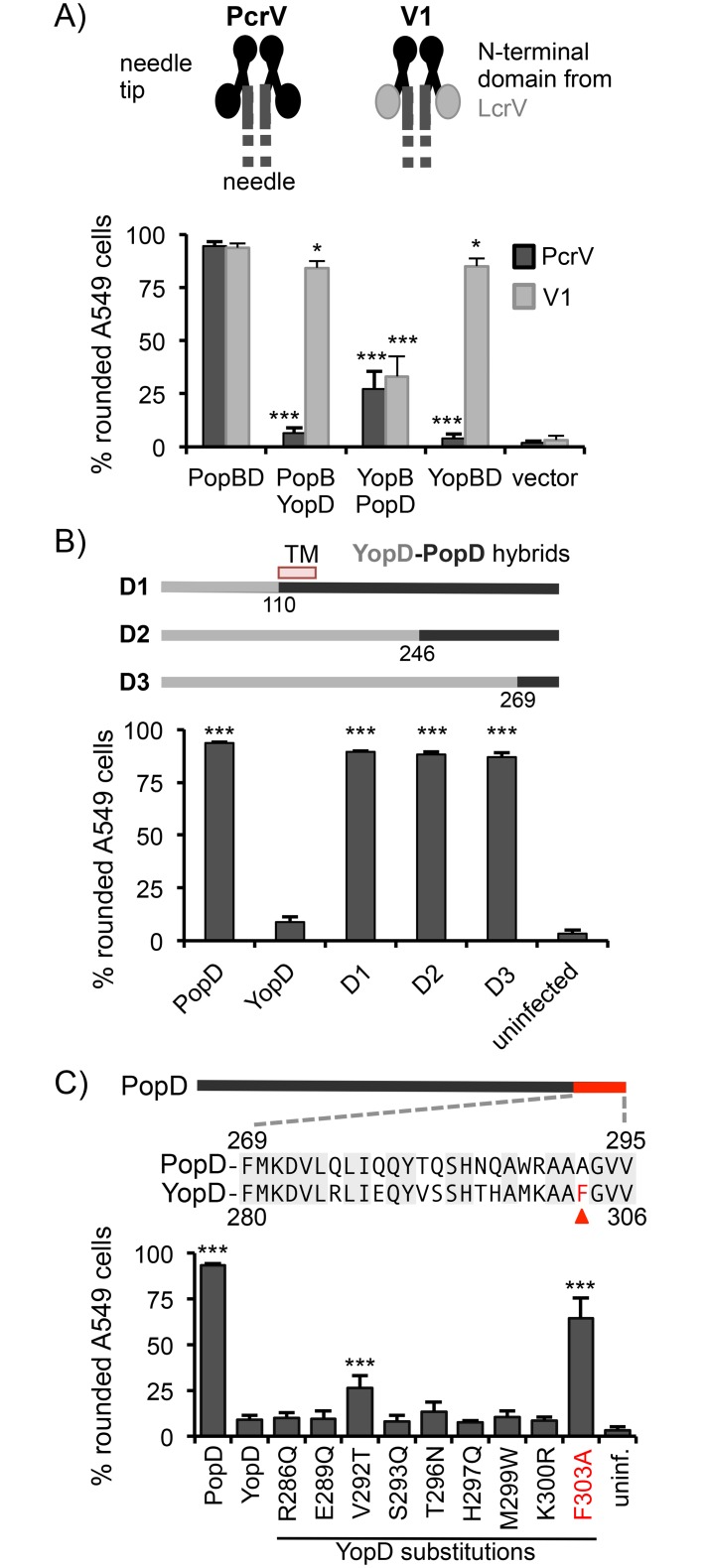
Critical translocator protein interactions. (A) Strain RP3624 (*pcrV*+ Δ*pcrHpopBD*, “PcrV”) or RP3714 (*lcrV-pcrV(V1)* “V1” Δ*pcrHpopBD*) were complemented with plasmids encoding the indicated combinations of translocator proteins. A549 cells were infected and delivery of ExoS into cells was monitored by assaying rounding of the cells due to ExoS-mediated depolymerization of actin. The diagram above the graph represents the two needle-tips, composed of either wild-type PcrV monomers, or hybrid V1 monomers, in which the N-terminal globular domain of PcrV was replaced with the corresponding domain of *Y*. *pseudotuberculosis* LcrV. Statistical significance was calculated by one-way ANOVA with Dunnett multiple comparisons test, comparing strains to the PopB/PopD producing wild type (PcrV+) control (* p<0.05, *** p< 0.001). In addition, no statistically significant difference was found when comparing the YopB/YopD producing strain to the PopB/YopD producing strain in the context of the V1 needle tip, whereas the difference when comparing the “V1” YopB/YopD strain to “PcrV+” PopB/YopD strain, or either of the YopB/PopD producing strains was significant (p<0.001). (B) Strain RP3624 (*pcrV*+ Δ*pcrHpopBD*, “PcrV”) was complemented with plasmids encoding *popB* paired with *popD*, *yopD*, or *yopD-popD* hybrid proteins. The hybrid proteins are diagrammed above the graph. The number of the first codon of *popD* is indicated below each *yopD-popD* junction. The ability of the strains to deliver ExoS into A549 cells was determined by monitoring rounding of epithelial cells. Statistical significance was calculated by one-way ANOVA with Dunnett multiple comparisons test, comparing strains to the PopB/YopD producing control strain (*** p< 0.001). (C) Strain RP3624 (*pcrV*+ Δ*pcrHpopBD*, “PcrV”) was complemented with plasmids encoding *popB* paired with *popD*, *yopD*, or mutants of *yopD*, in which individual codons had been replaced with the corresponding codon of *popD*. Translocation was monitored by assaying rounding of infected A549 cells. Statistical significance was calculated by one-way ANOVA with Dunnett multiple comparisons test, comparing strains to the PopB/YopD producing control strain (*** p< 0.001). Data in all panels represent the mean of at least four biological replicates with standard deviations.

Given the difficulty of studying translocon function *in vitro*, we employed a genetic approach to map translocator interactions that are critical for function. To this end we exploited the incompatibility between related translocator proteins. Previous work by others had shown that the translocator proteins of *P*. *aeruginosa* cannot substitute for the corresponding proteins in *Yersinia sp*., despite the overall similarity between these T3SSs. Specifically *popD* was unable to complement a *yopD* mutant of *Y*. *pseudotuberculosis* [12], and *pcrV* could not restore translocation in an *lcrV* mutant of *Y*. *eneterocolitica*. Interestingly, in the case of PcrV, function could be restored by exchanging the N-terminal globular domain of PcrV with that of LcrV, generating an LcrV-PcrV hybrid [27]. We recapitulated these data by producing the *Y*. *pseudotuberculosis* pore-forming translocator proteins, YopB and YopD, in *P*. *aeruginosa* and assaying their ability to restore translocation of effectors into A549 epithelial cells in a strain lacking the corresponding *P*. *aeruginosa* translocator proteins, PopB and PopD. Consistent with the data from *Y*. *enterocolitica*, YopB and YopD were unable to restore effector translocation unless the needle-tip protein PcrV was replaced by a hybrid protein in which the N-terminal domain of PcrV was replaced with the corresponding domain of LcrV (LcrV-PcrV hybrid “V1”)(Fig 1A). We next expanded on this observation by combining *P*. *aeruginosa* and *Y*. *pseudotuberculosis* translocators. Combining YopD with PopB, resulted in a translocon that was functional in the context of the V1 needle tip, but not wild-type PcrV (Fig 1A). These data indicate that PopB and YopD can form functional pores, but that YopD makes a critical contact with the N-terminal domain of LcrV that is broken when YopD is produced in the context of the *P*. *aeruginosa* needle-tip protein PcrV. The reciprocal combination, YopB and PopD, was non-functional regardless of the needle-tip, arguing that YopB and PopD are unable to form a functional translocon (Fig 1A). We conclude that combining *Yersinia* translocator proteins with *P*. *aeruginosa* translocator proteins breaks two interactions that are required for T3SS function: one between PopD and PcrV, the other between PopB and PopD.

### The C-terminus of PopD interacts with PcrV

We initially focused on the PopD-PcrV interaction since the mismatch incompatibility between YopD and PcrV resulted in the stronger defect in T3SS function. To map the region of PopD that interacts with PcrV, we generated YopD-PopD hybrid proteins in which portions of the C-terminal half of YopD were replaced by the corresponding region of PopD (S1A Fig). Strains producing these YopD-PopD hybrid proteins (D1-D3) in the context of the wild-type, PcrV, needle-tip were assayed for their ability to intoxicate epithelial cells (Fig 1B). YopD-PopD fusion proteins in which as little as the C-terminal 27 amino acids of YopD were replaced with the corresponding residues of PopD were able to fully substitute for PopD in this assay, arguing that it is the very C-terminus of PopD that has to interact with PcrV to allow intoxication of host cells. Indeed, substituting individual residues that differ between YopD and PopD in this region demonstrates that two of these differing residues contribute significantly to the loss of function when YopD is paired with PcrV (Fig 1C). Notably, the phenylalanine at position 303 of YopD is particularly deleterious for the interaction with PcrV, and a significant amount of T3SS function can be recovered by simply changing this residue to the corresponding alanine residue of PopD (Fig 1C).

**Fig 2:**
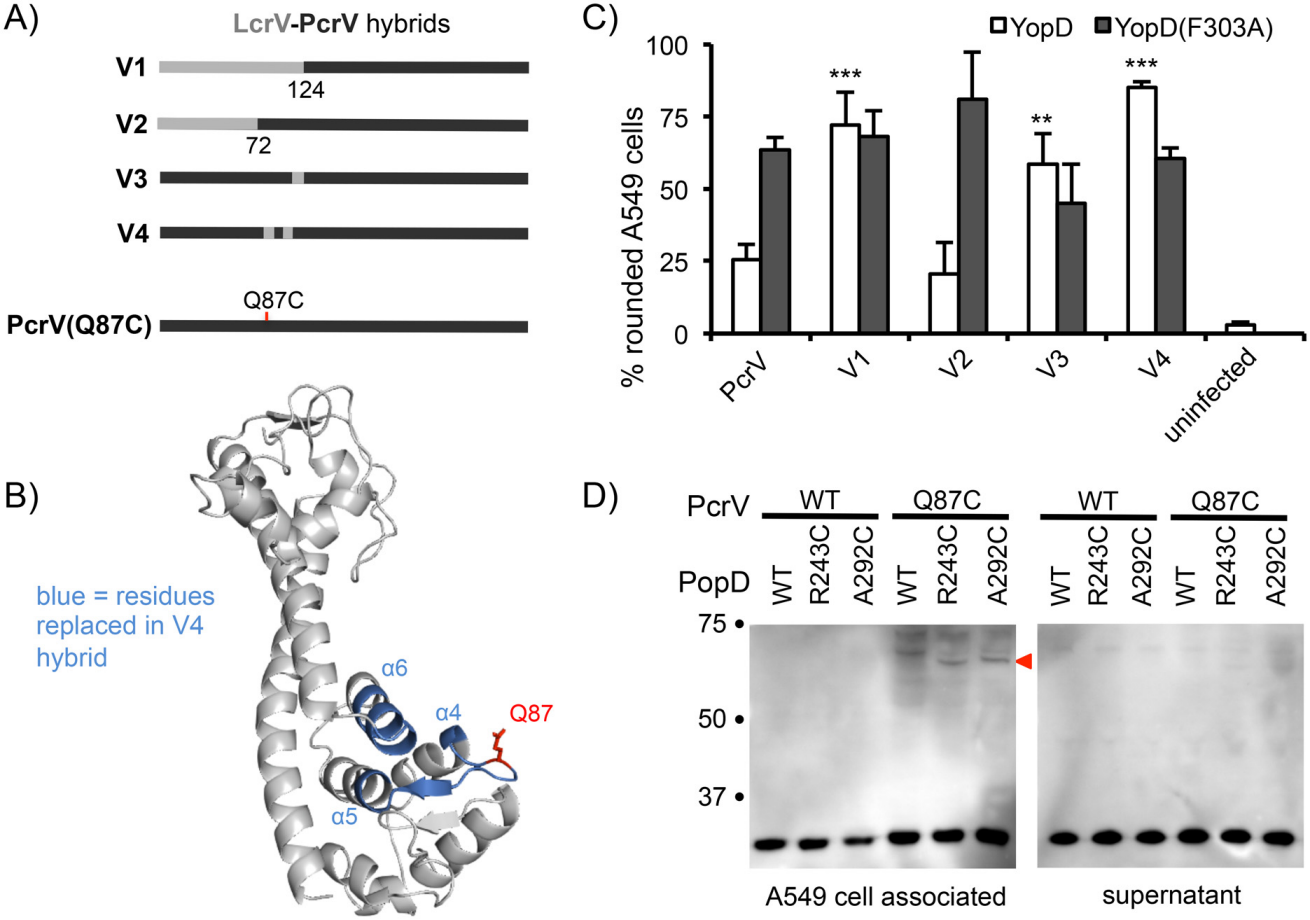
Mapping the region of PcrV that contacts the C-terminus of PopD. (A) Schematic representations of LcrV(light grey)-PcrV(dark grey) hybrids used in the experiment in panel C (see also S2 Fig). Residue numbers indicate the first PcrV residue of the hybrid protein. A schematic of the PcrV(Q87C) protein used in the crosslinking experiments is located at the bottom. (B) Threaded, structural model of PcrV. PcrV was threaded into the structure of LcrV [28] using Phyre [29]. The portions of alpha helices 4, 5, and 6 that are replaced with the corresponding LcrV residues in hybrid V4 are highlighted in blue. Residue Q87 is drawn in red, and the side chain is shown. (C) A549 cells were infected with *P*. *aeruginosa* strains producing either YopD or YopD(F303A) in the context of the indicated needle-tip protein. Delivery of effectors was monitored by rounding of A549 cells. The data are compiled from three independent experiments and shown as means with standard deviations. Statistical significance was calculated for the YopD-producing strains by one-way ANOVA with Dunnett multiple comparisons test, comparing strains to the wild-type PcrV producing control strain (** p<0.01, *** p< 0.001). (D) A549 cells were infected with *P*. *aeruginosa* strains RP2888 (*pcrV+* Δ*popD*) or RP6641 (*pcrV(Q87C) ΔpopD*) complemented with plasmids encoding *popD*, *popD(R243C)*, or *popD(A292C)*, as indicated, in the presence of the oxidant copper phenanthroline. Media from the infection, as well as cell-associated translocator proteins released by extracting the infected cells with Triton X-100, were separated by SDS-PAGE and probed for the presence of PcrV by western blot.

We similarly mapped the PopD-PcrV interaction by systematically replacing smaller portions of the N-terminal domain of PcrV with the corresponding regions of LcrV (Figs 2A, 2B and S2). The ability of these hybrid needle tips to support injection of host cells by bacteria producing YopD was assayed by monitoring intoxication of A549 epithelial cells (Fig 2C). YopD (F303A), which can function with PcrV and LcrV served as a control. These data indicate that the C-terminus of PopD has to contact a region of PcrV formed by α-helices 4, 5, and 6. This region was modeled by threading PcrV into the known structure of LcrV (Fig 2B). In order to demonstrate this contact directly, we substituted alanine 292 of PopD (corresponding to residue 303 of YopD) and residue Q87 of PcrV (Fig 2B) with cysteines to determine if these two residues can form a disulfide bond, linking PopD and PcrV. PopB, PopD, and PcrV are naturally devoid of cysteines. Infection of cells in the presence of copper as an oxidant resulted in formation of a PcrV-PopD disulfide bond which depended on the presence of both cysteines, demonstrating that the C-terminus of PopD indeed binds to PcrV at the positions indicated by our genetic mapping (Fig 2D). Notably, PopD(R243C), in which the cysteine lies outside of the C-terminal 27 amino acids that are responsible for the YopD-PcrV incompatibility, could also be crosslinked to PcrV(Q87C). While the latter result is consistent with the overall orientation of the translocator proteins and our assertion that the C-terminus of PopD interacts with the amino-terminal globular domain of PcrV, we cannot infer the exact manner in which the C-terminus of PopD binds to PcrV from these data. Instead, this result would argue that there is some mobility of the C-terminus of PopD relative to PcrV. Consistent with a function in translocation, the interactions could only be trapped in the context of the host cell, since the heterodimer could not be visualized in protein secreted into the culture supernatant.

### The PopD-PcrV interaction is required for triggering of effector secretion

Given that we can specifically break the PopD-PcrV contact by substituting the C-terminus of PopD with the corresponding region of YopD, we next sought to assign a specific function to this interaction. In principle, the interaction between PopD and PcrV could be involved in one of three aspects of translocon function: insertion of PopD into the host cell membrane, triggering of effector secretion, and delivery of effectors into the host cell (e.g. by forming a conduit between the bacterium and the host-cell).

**Fig 3:**
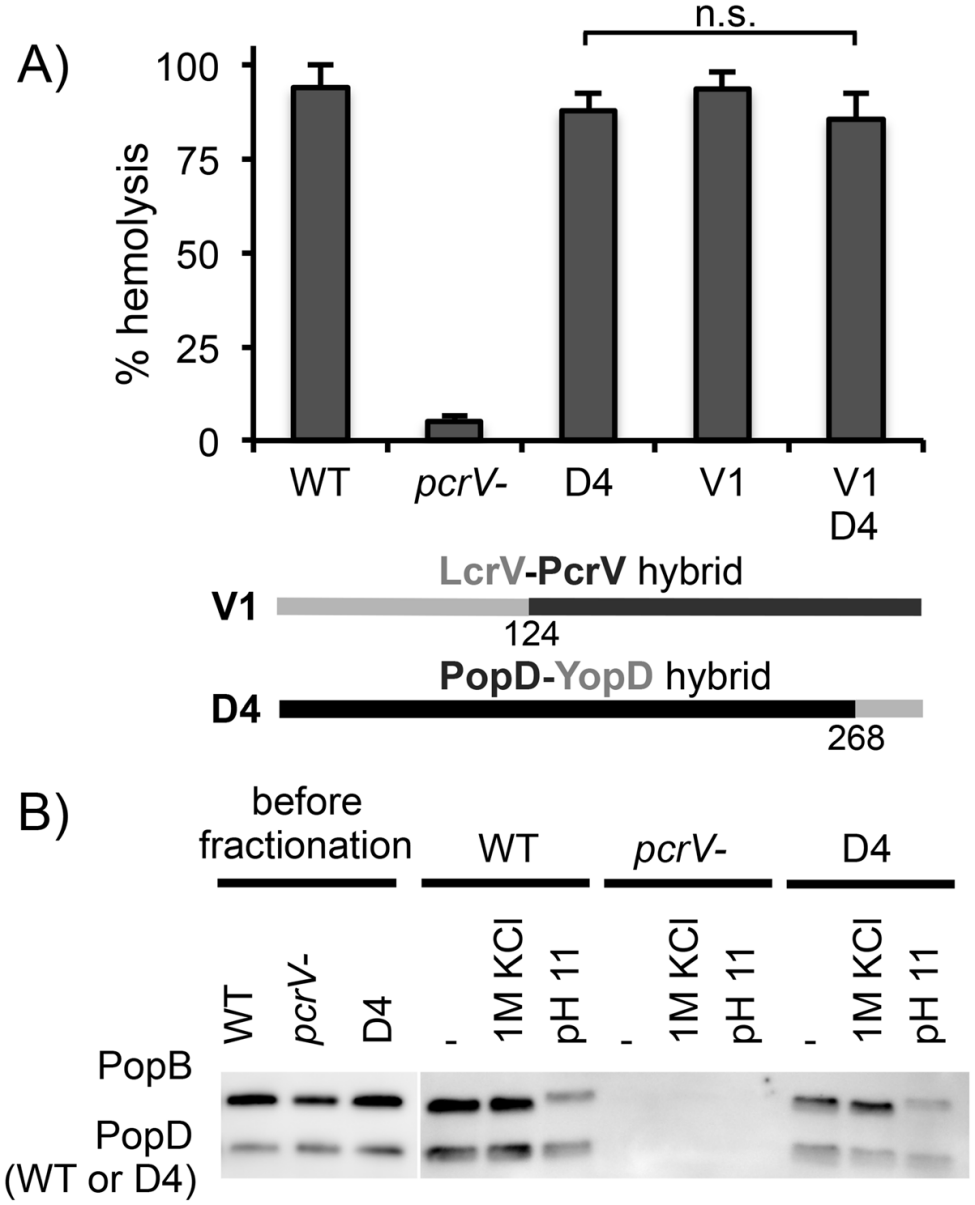
The PopD-PcrV interaction is not required for translocator insertion. (A) Red blood cells were infected for 1 hour with strains RP2349 (*popD+ pcrV+*, “WT”), RP9268 (Δ*pcrV2*, “*pcrV-*“), RP9270 (*pcrV+ popD-yopD(D4)*, “D4”), RP9272 (*popD+ lcrV-pcrV(V1)*, “V1”), or RP9274 (*lcrV-pcrV(V1) popD-yopD*(D4), ”V1 D4”). Hemolysis was assayed spectrophotometrically (OD415). Schematic representations of the LcrV-PcrV V1 hybrid, and PopD-YopD D4 hybrid are shown below the graph. Data are presented as mean of three independent experiments with standard deviation. Averages were compared by one-way ANOVA with Tukey post-hoc test using the GraphPad Prism software package. (B) Red blood cells were infected with strains RP2349 (*popD+ pcrV+*, “WT”), RP9268 (Δ*pcrV2*, “*pcrV-*“), or RP9270 (*pcrV+ popD-yopD(D4)*, “D4”). The cells were lysed through hypo-osmotic shock, and the plasma membranes were isolated by sucrose density gradient centrifugation. Membranes were pelleted by ultracentrifugation and, where indicated, washed once with 1M KCl or 1M NaCO3 (pH11) to remove peripherally associated proteins. Membrane samples were separated by SDS-PAGE. PopB and PopD were detected by western blot.

In order to distinguish between these three possibilities, we first examined the effect of breaking the PopD-PcrV contact on translocator insertion. Insertion of translocator proteins into red blood cells, and subsequent pore formation, results in hemolysis, which can be assayed spectrophotometrically [7]. To examine the effect of breaking the PopD-PcrV interaction on translocator insertion, we generated a strain that produces a hybrid PopD-YopD fusion protein (D4) in which the C-terminal 27 amino acids of PopD have been replaced with the corresponding residues of YopD. This strain is severely defective for delivery of effectors into host cells, unless produced in the context of the V1 needle tip (see below). Hemolysis in this assay is strictly T3SS-dependent, as illustrated by the fact that deletion of *pcrV* abrogates hemolysis entirely (Fig 3A). The strain producing the D4 hybrid translocator, on the other hand, promoted hemolysis to nearly the same level as cells producing PopD. Moreover, there was no statistical difference in hemolysis between a strain producing the D4 hybrid in the context of the wild-type PcrV needle tip (no interaction), as opposed to the hybrid V1 needle tip (restores interaction), arguing that translocon insertion is not significantly impaired. Indeed, assaying translocator insertion directly by isolating the RBC plasma membranes on sucrose density gradients illustrated that translocator insertion was not impaired (Fig 3B). Consistent with previously published data on PopB and PopD [9], the D4 hybrid was resistant to extraction of the membranes with high salt or high pH solutions, arguing that it is inserted into the membrane and not associated peripherally.

**Fig 4:**
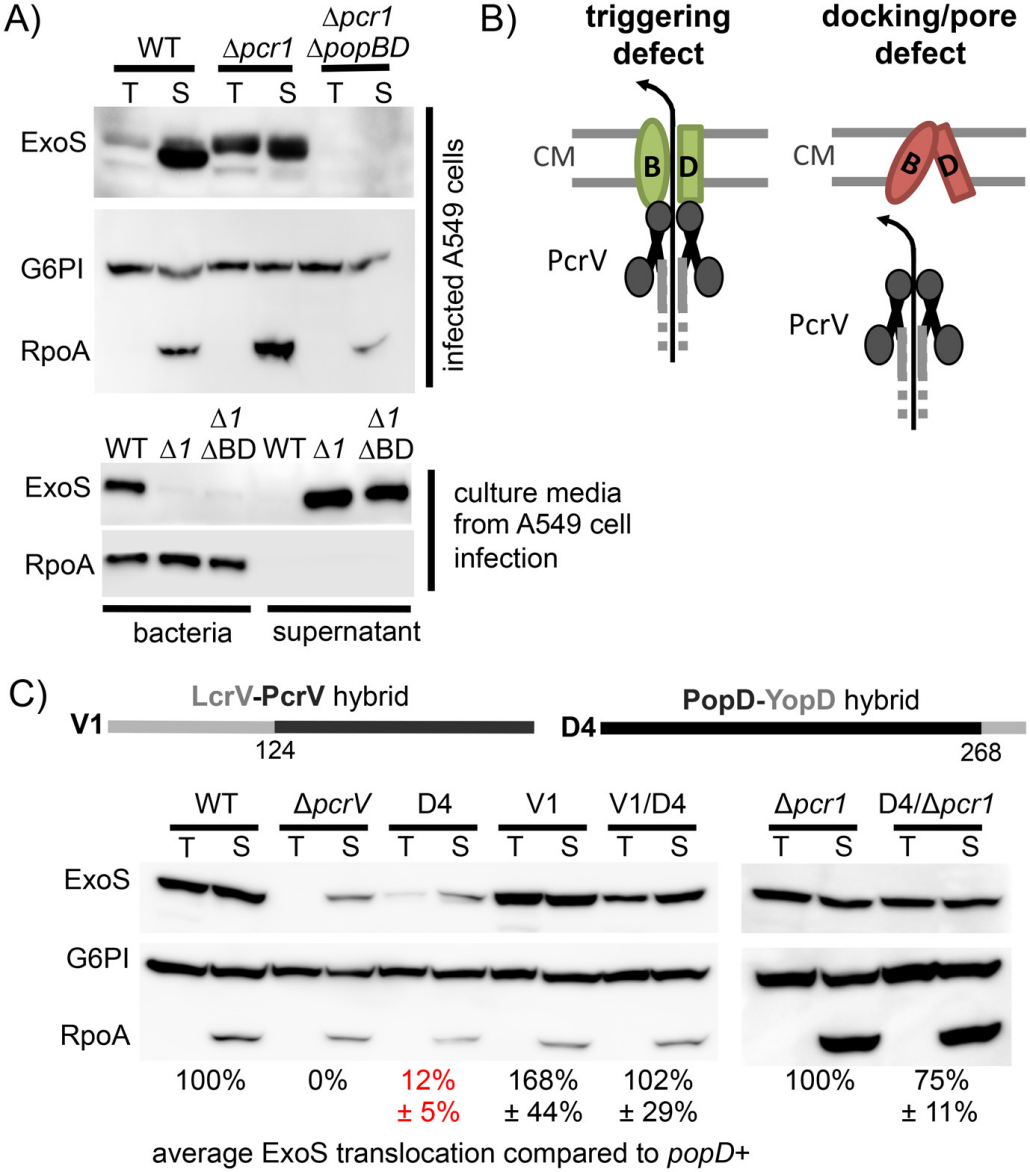
The PopD-PcrV interaction is required for triggering of effector secretion. (A) A549 cells were infected with strain RP2318 (“WT”), RP2996 (“Δ*pcr1*”), and RP6369 (, Δ*pcr1* Δ*pcrHpopBD*, “Δ*pcr1* Δ*popBD*”). Translocation of ExoS was monitored through differential lysis. Cells were either treated with Triton X-100 to release translocated protein (T), or SDS to solubilize both host cells and bacteria (S)(top). Supernatants were collected, and bacteria were pelleted to separate proteins secreted into the supernatant from bacteria (bottom). The presence of ExoS, RNA polymerase subunit α, (RpoA, bacterial cytoplasmic content), or glucose-6-phosphate isomerase (G6PI, host cell cytoplasmic content) was detected by Western blot. (B) Schematic outlining possible outcomes of combining production of the D4 PopD-YopD hybrid with a strain lacking *pcr1*. If the PopD-PcrV interaction disrupted by the D4 hybrid is required for triggering of effector secretion, then removing *pcr1* should restore translocation into host cells. Conversely, if the PopD-PcrV interaction is required for proper pore-formation or docking of the pore to the needle tip, then removing *pcr1* should fail to overcome the translocation defect. (C) A549 cells were infected with strains RP2349 (*pcrV+ popBD+*, “WT”), RP9268 (Δ*pcrV2*, *“*Δ*pcrV*”), RP9270 (*pcrV+ popD-yopD(D4)*, “D4”), RP9272 (*lcrV-pcrV(V1)*, “V1”), RP9274 (*lcrV-pcrV(V1) popD-yopD(D4)*, “V1 D4”), RP9266 (Δ*pcr1* “Δ*pcr1*”), or RP9276 (Δ*pcr1 popD-yopD(D4)*, “Δ*pcr1* D4”). Translocation of ExoS into host cells was monitored by differential lysis as outlined in (A). Translocation was quantified by normalizing ExoS to G6PI in the Triton X-100 sample, which was subsequently compared to the level of translocated ExoS in the corresponding WT sample (Δ*pcrV*, D4, V1, and V1 D4 were compared to WT; D4 Δ*pcr1* was compared to the Δ*pcr1* strain). Data are reported as the mean of at least three biological replicates with standard deviations. Statistical significance was calculated by one-way ANOVA with Dunnett multiple comparisons test, comparing the D4, V1/D4 and D4 Δ*pcr1* samples to the corresponding wild-type. Of these strains, only infection with the D4 mutant strain resulted in a statistically significant defect in translocation (p<0.01).

Based on these results, the defect incurred by breaking the PopD-PcrV interaction could therefore be either at the level of triggering of effector secretion, or delivery of effectors into the host cell (docking/conduit formation). We distinguished between these two possibilities by making use of a mutation that abrogates the need for a specific trigger for effector secretion [30, 31]. Deleting *pcr1*, encoding a component of the PopN complex, which prevents effector secretion before cell contact, results in secretion of effector proteins in the absence of host cell contact [32, 33]. Notably, a *pcr1* mutant is still able to inject effectors into host cells, an activity that relies on a functional translocon (Fig 4A). Therefore, if breaking the PopD-PcrV contact blocks triggering, removing *pcr1* should restore effector translocation, since the system no longer needs a specific trigger to commence secreting effectors. Conversely, if the PopD-PcrV contact is need for conduit formation, then removing *pcr1* should not correct the defect incurred by breaking this interaction (Fig 4B). To determine which of these two possibilities is the case we assayed injection of ExoS into epithelial cells directly. Here we relied on a strain that produces a mutant version of ExoS in which both active sites have been inactivated by point mutation. This strain increases the signal in the assay, since ExoS feedback inhibits its own translocation thereby severely limiting the amount of the wild-type protein injected [17]. As can be seen in Fig 4C, breaking the PopD-PcrV contact severely impairs translocation of ExoS into host cells. Restoring the contact by also producing the V1 needle tip restores translocation, indicating that the defect in translocation in strains producing the D4 PopD-YopD hybrid is due to the lost PopD-PcrV interaction. Importantly, in a strain lacking *pcr1* translocation of ExoS by the strain producing the D4 hybrid is restored to near wild type levels, arguing that the PopD-PcrV contact is specifically required for triggering of effector secretion (Figs 4C and S3).

### The translocon actively senses host cell contact

**Fig 5:**
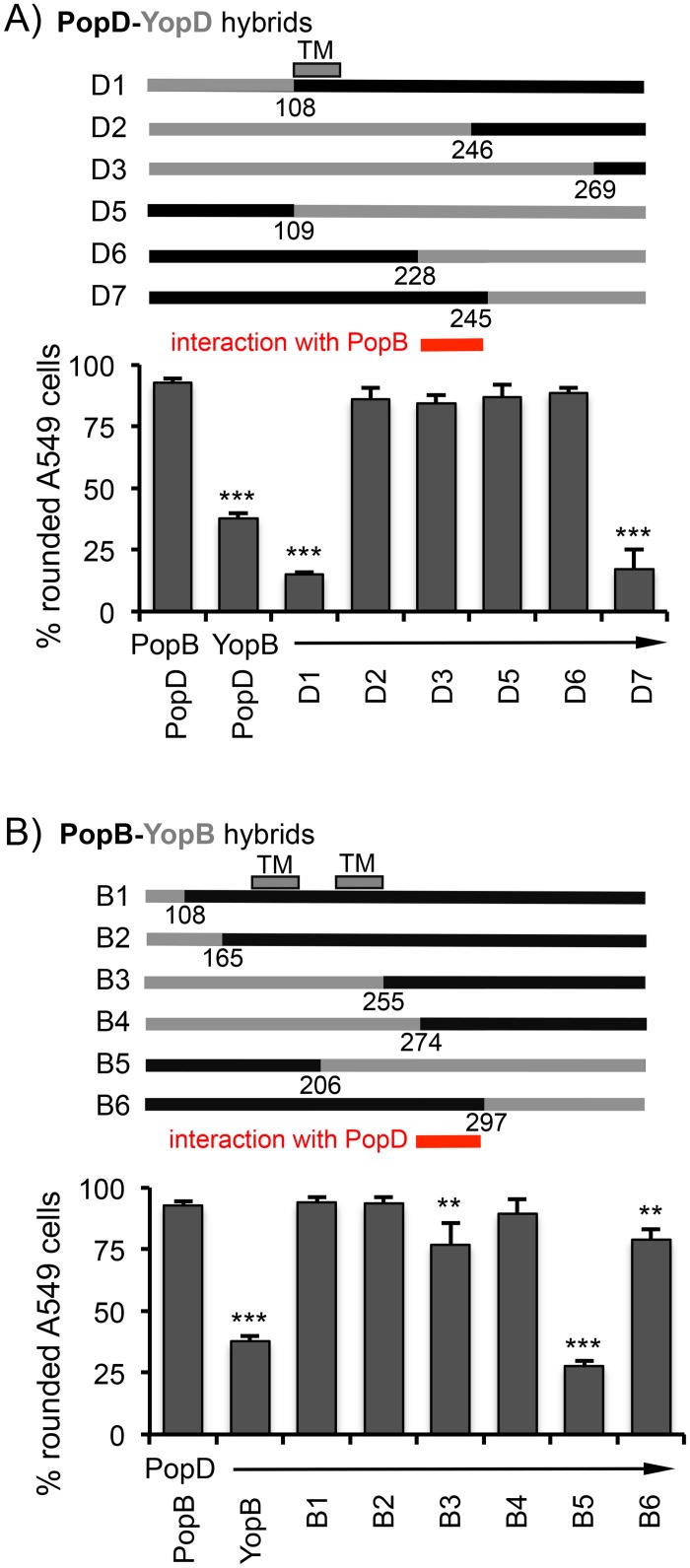
Mapping of a contact between PopB and PopD that is needed for translocon function. Strain RP3714 (*lcrV-pcrV(V1)* Δ*pcrHpopBD*), producing the indicated combinations of pore-forming translocator proteins, was used to infect A549 epithelial cells. Translocation of ExoS was assayed by monitoring cell rounding due to actin depolymerization. Hybrid proteins used to map the interaction between PopB and PopD are indicated above each graph. Numbers below each junction represent the first, or last amino acid, of the PopD (A) or PopB (B) portion of the hybrid translocator protein. Statistical significance was calculated by one-way ANOVA with Dunnett multiple comparisons test, comparing samples to the PopB/PopD producing wild type control (** p<0.01, *** p< 0.001).

We next set out to map the critical contact between PopB and PopD that is disrupted by pairing PopD with YopB. We again generated strains producing PopD-YopD and PopB-YopB hybrid proteins (S1 Fig), paired with YopB and PopD, respectively. Restoration of T3SS function was assayed by monitoring the ability to intoxicate epithelial cells (Fig 5). These experiments allowed us to narrow down the region of interaction to residues 228–245 of PopD and 274–297 of PopB. However, we were unable to assign the bulk of the defect in the YopB-PopD interaction to a specific residue, as had been the case for the PopD-PcrV interaction. Instead, the defect appears to be due to numerous small incompatibilities that are difficult to map genetically.

**Fig 6:**
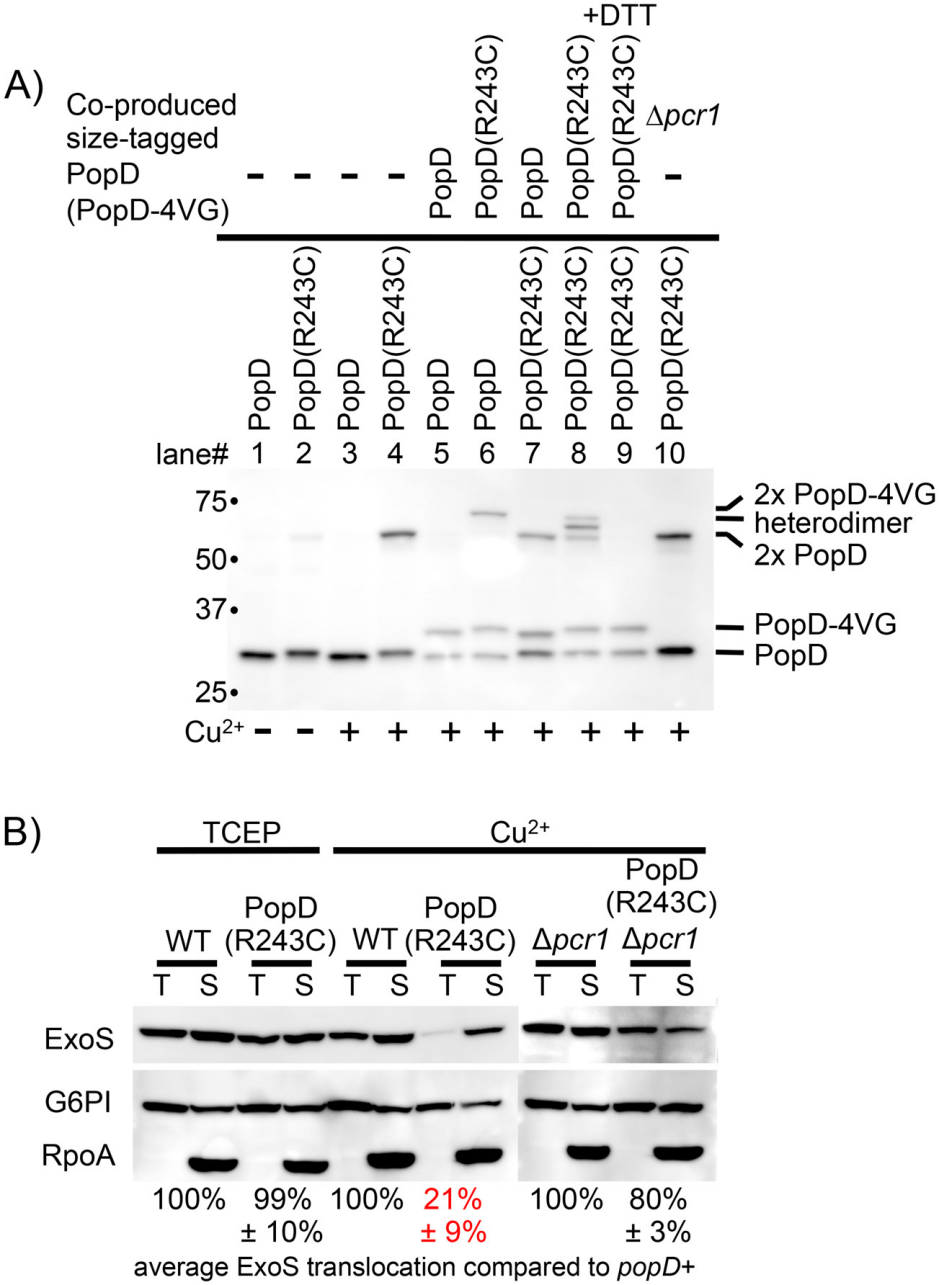
Tethering the PopD dimer blocks triggering of effector secretion. (A) Dimer formation was assayed by infecting A549 cells with strain RP2349 (*popD+*), RP8136 (*popD(R243C)*), or strain RP8766 (*popD(R243C)* Δ*pcr1*). Where indicated a 4xVSV-G tagged version of PopD or PopD(R243C) was produced from a plasmid. Expression was induced through the addition of 200μM IPTG. The infection was carried out in the presence of the oxidant copper-phenanthroline, where indicated. At the end of the incubation period free cysteines were alkylated through the addition of iodoacetamide, cells were washed with 1M KCl to remove peripherally associated proteins, and translocators inserted into host cell membranes were extracted with Triton X-100. Membrane extracts were separated by SDS-PAGE and probed for the presence of PopD by Western blot. (B) A549 cells were infected with strains RP2349 (*popD+*, “WT”), RP8136 (*popD(R243C)*), or strain RP8766 (*popD(R243C)* Δ*pcr1*). The infections were carried out in the presence of the reductant TCEP, or the oxidant copper phenanthroline as indicated. Infected cells were extracted with Triton X-100 (T), or SDS (S), and protein samples were separated by SDS-PAGE. ExoS, G6PI, and RpoA were detected by Western blot. Translocation was quantified by normalizing ExoS to G6PI in the Triton X-100 sample, which was subsequently compared to the level of translocated ExoS in the corresponding WT sample. Data are reported as the mean of at least three biological replicates with standard deviations. Statistical significance was calculated by unpaired t-test with Welch’s correction, comparing the PopD(R243C) sample to the corresponding wild-type. In the presence of copper, translocation was significantly reduced in the PopD(R243C) producing strain compared to wild-type (p<0.01 for both). Translocation, in the presence of copper, was significantly increased in the *popD(R243C)* Δ*pcr1* strain, compared to the *popD(R243C)* mutant strain (p<0.01), but still significantly below the *popD+* Δ*pcr1* parent (p<0.01).

In the course of attempting to introduce cysteines into PopB and PopD to crosslink the two proteins, we found that both PopB and PopD form dimers, which can be trapped by an intermolecular disulfide bond (Figs 6A and S4A, lane 4). In order to demonstrate that these higher molecular weight species represent dimers of PopB and PopD, respectively, we co-expressed functional, size-tagged versions of either translocator, in which four copies of the VSV-G-epitope had been inserted at a neutral site. Insertion of the VSV-G tags allowed us to distinguish the tagged version of the translocator from that expressed by the untagged chromosomal gene (Figs 6A and S4A, lanes 5–9). Indeed, if PopD(R243C) and the size-tagged version, PopD(R243C)-4VG, were co-expressed in the presence of copper, we detected three higher molecular weight complexes, indicating the formation of homodimers of either protein, as well as a heterodimer of intermediate molecular weight (Fig 6A, lane 8). The latter species would not be expected if PopD were disulfide bonding to a different protein. Reduction of the sample through the addition of DTT dissolved the higher molecular weight complexes (Fig 6A, lane 9), demonstrating that they are tethered through a disulfide bond. The same was true for PopB(A280C) (S4 Fig).

Since we can use the PopD(R243C) disulfide bond to tether the PopD dimer, we next asked whether linking the two PopD molecules interferes with translocon function. Indeed, tethering the PopD dimer at position 243 dramatically reduced the amount of ExoS translocated into epithelial cells (Fig 6B). Importantly, this defect was not evident when the experiment was performed in the presence of the membrane-impermeant reductant TCEP, demonstrating that PopD(R243C) is not defective *per se*, rather formation of the disulfide bond prevents effector translocation. Tethering PopB at position A280, on the other hand, only had a minor effect on translocation (S4 Fig), demonstrating that the defect in translocation is not an artifact of the growth conditions. We next removed *pcr1* to test whether the defect in translocation is due to a defect in triggering of effector secretion, or due to malformation of the translocon, preventing effector delivery. As for the PopD-PcrV interaction above, removing *pcr1* restored effector translocation in the presence of copper by the strain producing PopD(R243C). Deleting *pcr1* does not interfere with dimer formation (Fig 6A), arguing that tethering the PopD-dimer at position 243 specifically interferes with triggering of effector secretion. By extension, these data demonstrate that the translocon is actively involved in sensing host-cell contact. Tethering the PopD dimer presumably blocks a conformational change that is induced by host-cell contact and is transmitted to the base of the apparatus by an interaction with the needle-tip.

## Discussion

We exploited the incompatibility between the related translocator proteins of *P*. *aeruginosa* and *Y*. *pseudotuberculosis* to map protein-protein contacts among translocator proteins that are critical for translocon function. Our analysis indicated that expressing *Y*. *pseudotuberculosis* translocator proteins in *P*. *aeruginosa* disrupts two interactions: one between PopD and PcrV, the second between PopB and PopD. The PopD-PcrV interaction is specifically required for triggering of effector secretion. Moreover, our analysis indicates that triggering of effector secretion likely involves a conformational change in the translocation pore, which is then transmitted to the needle tip. Our data also lend insight into the overall organization of the translocon. Both pore-forming translocator proteins form dimers, and the location of the contacts we mapped allow us to orient PopB, PopD, and PcrV relative to one another.

**Fig 7:**
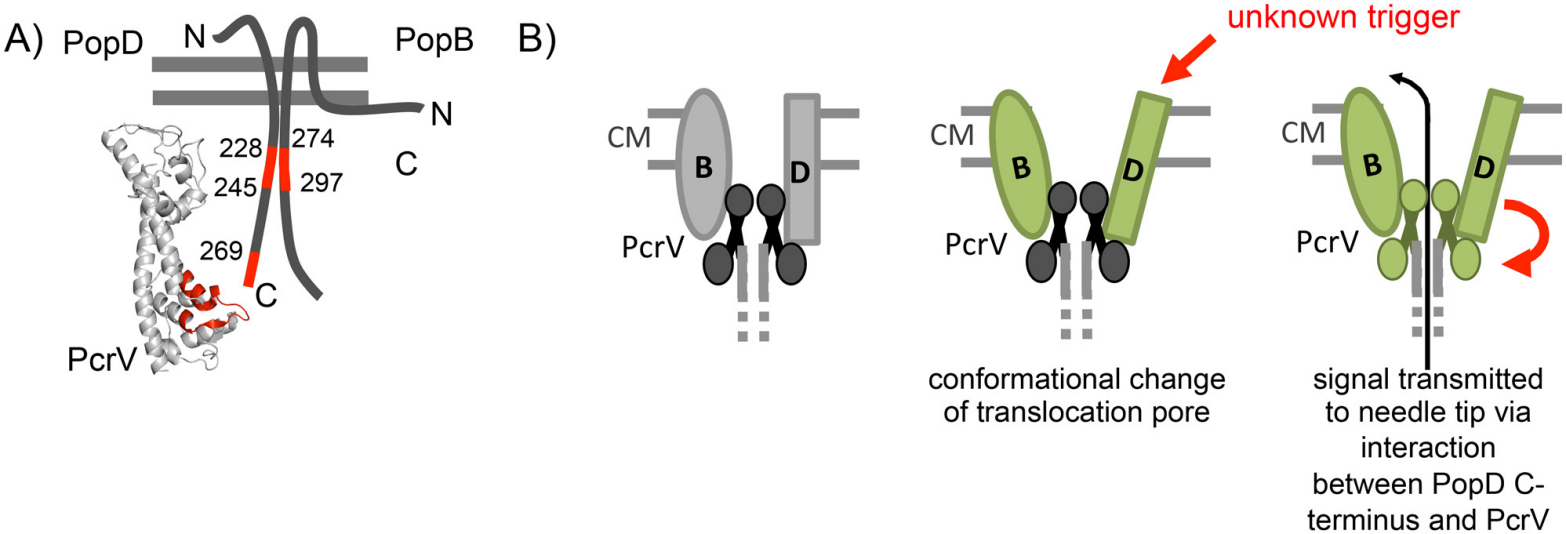
Models of translocon organization and function. (A) Model of interactions identified in this study. PopD (aa228-245) interacts with PopB (aa274-297). PopD(269–295) interacts with the N-terminal globular domain of PcrV. PcrV is represented as a model based on the structure of *Y*. *pestis* LcrV [28], generated using Phyre folding server [29]. The region that binds to the C-terminus of PopD is indicated in red. The host-cell plasma membrane is indicated as a grey double line. (B) Once the translocator proteins have been inserted (left), an as yet unknown trigger effects a conformational change in the pore formed by PopB and PopD (middle). This signal is transmitted to the needle tip via an interaction between the PopD C-terminus and PcrV (right).

The organization of the translocation pore has been difficult to study. In part, this is due to the fact that it has not been possible to isolate translocation pores assembled in the context of an infection. Export of translocators before cell contact [17], and presumably non-specific binding to cell surfaces, has further muddled the analysis of the topology of inserted translocators. For example, three orientations have been proposed for the translocator equivalent to PopD in the *P*. *aeruginosa* system: N-terminus in/C-terminus out, N-terminus out/C-terminus in and both N and C-terminus out [34–36]. By mapping contacts that are required for translocon function, we can sidestep these difficulties since any protein-protein interaction we identify has to occur either during assembly of, or in the context of the fully formed translocon. Accordingly, our data indicate that the C-terminus of PopD faces the extracellular milieu, where it contacts the PcrV needle tip (Fig 7A). Our data are also consistent with the recently published topology of PopB, whereby PopB is proposed to insert into the host cell plasma membrane with both the N- and C-terminus facing the extracellular milieu [37]. It seems likely that PopD is inserted into the host cell plasma membrane with the N-terminus facing the cytoplasm rather than being attached peripherally since PopD is resistant to extraction by high salt or high pH solutions [9] (Fig 3B). This has been disputed due to the difficulty of inserting PopD into artificial membranes *in vitro*, leading to the proposal that only PopB inserts to form the pore, while PopD serves to connect PopB to the needle-tip. [38]. A possible explanation for this discrepancy is the fact that insertion of the pore-forming translocator proteins, when delivered by the bacterium, is facilitated by the needle-tip [9, 11, 27], which prompted Cornelis and co-workers to propose that the needle-tip functions as a scaffold for the assembly of the translocation pore [8, 27].

Triggering of effector secretion on cell contact is a hallmark of T3SSs, however the mechanism of triggering remains enigmatic. Up to this point, the role of the translocon in triggering effector secretion has been unclear. In the case of the *Shigella flexneri* T3SS it was proposed that insertion of one of the translocator proteins, IpaB, results in recruitment of the second pore-forming translocator protein, IpaC, which in turn allows formation of the translocation pore and subsequent effector export [39]. While several triggers have been proposed, for example exposure of the needle to the low-calcium environment of the host cell, or changes in pH [22, 23], it is unclear from these data whether the translocon has a passive role in triggering of effector secretion (e.g. by establishing the pore which then allows another part of the secretion needle to respond to a chemical property of the connected compartment), or whether the translocon actively senses host cell contact. Our data indicate that the latter is the case. Tethering the dimer of PopD at position 243 by introducing a disulfide bond specifically interfered with triggering of effector secretion, but not pore-formation or injection of effectors. These data argue that the translocation pore has to undergo a conformational change to trigger effector export, which is prevented by the disulfide bond at position 243.

Our data indicate that the C-terminus of PopD has to interact with PcrV in order to transmit the signal to commence effector secretion. Early data using the *Y*. *pseudotuberculosis* system indicated that the C-terminus of YopD is important for translocation. Small deletions of residues 278–292, or 293–305, blocked effector translocation [40, 41], however, these deletions also impacted assays of pore-formation, such as translocon-mediated hemolysis, arguing that the deletion mutants had lost the ability to form a functional translocon altogether [41]. Subsequent analysis of point mutations in a putative amphipathic α-helix located near the C-terminus of YopD (residues 278–292) revealed that a subset of these mutations had a reduced ability to bind to the needle-tip protein LcrV in a pull-down assay [42]. Several of these mutants had also lost the ability to deliver effectors into HeLa cells, which led the authors to propose that the YopD-LcrV interaction is needed for function. Our analysis is consistent with- and significantly extends these findings. First, we were able to map the point of contact of PopD on PcrV. Moreover, we demonstrated through direct crosslinking, that this interaction occurs specifically at the cell surface upon translocon assembly (as noted in the introduction, PcrV fails to bind to PopD *in vitro*, [18, 21]). Finally, our data indicate that the interaction of the extreme C-terminus of PopD with PcrV (which differs slightly from the region analyzed by Costa et al.) is specifically required for triggering of effector secretion. The proposed role of the N-terminal domain of PcrV in signal transduction is further bolstered by a previously published linker insertion mutagenesis analysis of PcrV function. Here, three insertions in the N-terminal domain (after amino acids 44, 52, and 64 of PcrV) resulted in proteins that still supported translocation of effectors into host cells, but resulted in deregulated effector secretion [43]. PcrV prevents effector secretion by constraining the T3SS in an effector secretion off conformation [44]. These insertion mutants have specifically lost the ability to constrain the apparatus in the effector secretion “off” conformation. By extension, we would propose that the triggering conformational change in PopD similarly relieves the constraint PcrV imposes on effector secretion through its interaction with the N-terminal domain of PcrV. A similar constraint on effector secretion by the needle-tip complex has been proposed in *S*. *flexneri*. Here the tip complex is composed of two proteins, IpaD and IpaB [39]. A short, three amino acid C-terminal truncation of IpaB results in a protein that still associates with the needle-tip, but has lost the ability to prevent effector secretion before cell contact [45]. The model is also compatible with known needle mutations that trigger effector secretion in the absence of cell contact, as well as mutations throughout the basal body that similarly result in premature secretion of effectors [46–49].

Our data identifies a signal transduction pathway (Fig 7B), whereby an as yet unknown trigger effects a conformational change in the translocation pore. This structural change is then transmitted to the needle tip via an interaction between PopD and PcrV, and from there, through a conformational change in the needle, to the base of the apparatus.

## Materials and Methods

### Bacterial strains, cells, and growth conditions

Bacterial strains and plasmids used in this study are listed in S1 Table. *E*. *coli* strains were grown in LB medium (10g tryptone, 5g yeast extract, 10g NaCl per liter), supplemented with 15μg/ml gentamicin when necessary. *P*. *aeruginosa* strains were grown on “high salt” LB (10g/L tryptone, 5g/L yeast extract, 200mM NaCl, 5mM MgCl2, 0.5mM CaCl2), supplemented with 30μg/ml of gentamicin when necessary. Assembly of the T3SS is controlled by the osmolarity of the medium. Use of “high salt” LB allows for consistent production of the T3SS under laboratory conditions [50]. Production of translocator proteins was induced through the addition of IPTG to the growth medium.

A549 cells (American Type Culture Collection, Cat. #CCL-185) were grown in RPMI1640 medium supplemented with 10% fetal bovine serum (RP10) at 37°C in a 5% CO2 atmosphere. Cells were maintained in the presence of penicillin and streptomycin. Before experiments, cells to be infected were washed 1x with Dulbecco’s phosphate buffered saline (DPBS), and the medium was exchanged with RP10 lacking antibiotics.

### Plasmid and strain construction

Mutations were introduced into the chromosome of strains through allelic exchange as described previously [51]. Plasmids were constructed using standard molecular biological techniques. The indicated translocator (or portion of a translocator) was amplified by polymerase chain reaction using the primers listed in S1 Table. *Y*. *pseudotuberculosis* YPIII DNA was used as template for the amplification of amplification of *yopD*, *yopB*, and *lcrV*. Hybrid translocators were generated using splicing by overlap extension (SOE) PCR. PCR products were cut with the appropriate restriction enzymes (listed in S2 Table) and ligating into plasmids pEXG2 (allelic exchange vector) or pPSV37 (plasmid that can replicate in *P*. *aeruginosa* with *lacUV5* promoter and *lacIq*).

### Cytotoxicity

*P*. *aeruginosa* strains were grown to mid-logarithmic phase in “High salt” LB, pelleted and resuspended in PBS-MC [Dulbeccos’s phosphate buffered saline, Invitrogen Cat# 14190–144, supplemented with 5mM MgCl_2_ and 0.5mM CaCl_2_ (final concentrations)]. A549 epithelial cells were infected at an MOI of 10 for 2–4 hours. Where necessary, 100μM IPTG was added to the media to induce translocator gene expression from plasmid constructs. Following the infection period, the media was removed, and the cells were fixed using 3.7% formaldehyde for 15 min. Rounded versus flat cells were counted by low-power phase contrast microscopy. Data are reported as mean of three biological replicates with standard deviation.

### Translocation

*P*. *aeruginosa* strains were grown to mid-logarithmic phase in “High salt” LB, pelleted and resuspended in PBS-MC. A549 epithelial cells were infected with *P*. *aeruginosa* strains at an MOI of 25 for 2 hours. The cells were then washed three times with PBS-MC, and rinsed with 1ml of 250 μg/mL proteinase K in PBS-MC. The protease solution was then removed, and the cells were incubated at room temperature for 15 minutes to digest extracellular protein. The protease-treated cells were resuspended in 1ml of PBS-MC with 2mM phenylmethylsulfonyl fluoride (PMSF), and pelleted (3 minutes, 5000 rpm). The cells were resuspended in 300μL of PBS-MC with 0.5% Triton X-100 and incubated on ice for 15 minutes. 150μL of the cell suspension were removed and mixed with 50μl of 4x SDS sample buffer (SDS sample). The remaining cells were pelleted, and 150μL of supernatant were removed and combined with 50μL of 4x SDS sample buffer (Triton solubilized fraction). The samples were separated by SDS-PAGE and analyzed by Western blot. Membranes were probed with antibodies directed against ExoS, RpoA (Neoclone) and either human glucose-6-phoshate isomerase (G6PI, Santa Cruz Biotechnology Inc.), actin (Developmental Studies Hybridoma bank), or tubulin (Santa Cruz Biotechnology Inc.). Antibodies were detected using a horseradish peroxidase labeled secondary antibody, and WesternBright Quantum reagent and imaged using a GE ImageQuant LAS4000 imaging system. Protein levels were quantitated using ImageJ, and ExoS levels were normalized to G6PI (or actin, or tubulin)-levels.

### Crosslinking

Pseudomonas strains producing cysteine mutants of PopD, PopB, or PcrV (as indicated) were grown to mid-logarithmic phase in “High salt” LB with 200μM IPTG, pelleted and resuspended in PBS-MC. A549 epithelial cells were infected for 2 hours in the presence of 200μM IPTG and either 25 μM copper phenathroline or 1mM TCEP. After infection cells were washed with 25 mM iodoacetamide for 3 min, to alkylate free cysteine residues. Cells were then washed once with high salt PBS (PBS-MC + 1 M KCl) before the cells were scraped up in 1ml of PBS-MC with 2mM phenylmethylsulfonyl fluoride (PMSF), and pelleted (3 minutes, 5000 rpm). The cells were resuspended in 45μL of PBS-MC with 0.5% Triton X-100 and incubated on ice for 15 minutes. The cells were pelleted, and 45μL of supernatant were removed and combined with 15μL of 4x SDS sample buffer without DTT. Samples were analyzed by Western blot. Membranes were probed for the presence of PcrV, PopB, or PopD using affinity-purified antibodies.

### Hemolysis

The protocol we used was based on the assay published by Blocker *et al*. [7]. Sheep red blood cells (Quadfive) were washed three times with PBS-MC, and resuspended in RPMI without phenol red or FBS to a concentration of 5 x10^8 cells/mL. Bacterial cultures were diluted 1:250 from overnight cultures into high salt LB and allowed to grow to mid-log phase. Bacterial cultures were spun down and resuspended in PBS MC, the OD600 was measured and bacteria was resuspended to a concentration of 2.5 x 10^9 cells/mL. Bacteria and red blood cells were mixed 1:1 (MOI of 5) in a v-bottomed 96-well plate. Samples were centrifuged at 2000 g for 10 minutes and then incubated for 1 hour at 37°C. The samples were then resuspended and centrifuged at 2000 g for 10 minutes before collecting 100 μL of supernatant. Supernatants were placed in a clean flat-bottomed 96-well plate and then hemoglobin release was calculated by measuring absorbance at 415 nm. Base line lysis was determined by red blood cells mixed and incubated with PBS MC. Percent lysis was determined by comparing samples to 100% lysis using 0.1% SDS.

### RBC Membrane Isolation

Red blood cells and bacterial samples were prepared as noted for the hemolysis assay. Samples were prepared by mixing 1.25 mL of a 8x10^8 cells/mL red blood cell suspension with 250 μL of a 2x10^10 cells/mL bacterial suspension in the presence of 1X cOmplete protease inhibitor. They were then centrifuged at 2000 g for 10 minutes, and incubated at 37°C for 1 hour. Samples were resuspended and centrifuged at 2000g for 10 min before lysing with distilled water on ice for 10 minutes. Intact cells were centrifuged out at 5000 rpm for 10 minutes. The supernatant containing the lysed membranes were removed. A 30μl aliquot was removed and mixed with 10μL of 4xSDS sample buffer to serve as input control. A sucrose gradient was formed by layering 0.5 mL 50%, 1 mL 41%, and 1 mL 20% sucrose in 20 mM HEPES pH7.6 1mM EDTA. Red blood cell membranes were isolated by separating the supernatant on a sucrose gradient (Beckman Coulter Optima Max-XP ultracentrifuge; MLS50 rotor; 34,407rpm (95,000 rcf) for 2 hours), and collecting the membranes at the interface between the 41% and 20% sucrose phases. Samples were split into three parts. One part was diluted with ~10x volume of 10 mM HEPES pH7.6 1mM EDTA buffer, one with 1M KCl, and the last part with 1 M Na_2_CO_3_. The membranes were pelleted by ultracentrifugation (TLA100.3 rotor, 40,000 RPM for 1h), resuspended in 50μL 1X SDS sample buffer, and boiled at 95°C for 10 min before. Samples were desalted using Zebra spin desalting columns (Thermo) and mixed with 4xSDS sample buffer (80μl final volume). Samples were analyzed by Western blot (input samples were diluted 1:2 and 10μl were loaded on the gels, 20μl of the membrane preps were loaded). Membranes were probed with antibodies directed against PopB and PopD.

## Supporting Information

S1 Fig PopD/YopD and PopB/YopB alignments. A) PopD (Genbank accession AAG05098) and YopD (Genbank accession AAA72322) were aligned using ClustalW. The amino acid sequences are 39% identical (58% similar). Identical residues are shaded dark grey, similar residues in light grey. Residue numbers next to the alignment rows correspond to the amino acid number of the *P*. *aeruginosa* translocator. Predicted transmembrane domains (TM) are indicated by olive lines above the corresponding amino-acid sequence. Residue A292 of PopD, which corresponds to F303 in YopD, is highlighted since it, when substituted with cysteine, allowed PopD to be disulfide bonded to PcrV(Q87C). Fusion junctions of hybrid proteins used in Figs 1 and 5 are highlighted in red. B) PopB (Genbank accession AAG05097) and YopB (Genbank accession AAA72321) were aligned using ClustalW. The proteins are 40% identical (65% similar). Fusion junctions of hybrid proteins in Fig 5 are highlighted in red.
(TIF)

S2 Fig PcrV/LcrV alignment. PcrV (*P*. *aeruginosa* PAO1, Genbank accession AAG05095) and LcrV (*Y*. *pseudotuberculosis* YPIII, Genbank accession AAA27645) were aligned using ClustalW in MacVector (MacVector Inc.). Identical residues are shaded dark grey, similar amino acids are shaded light grey. Overall, PcrV and LcrV are 37% identical (54% similar). α- helices 4, 5, and 6, based on structural modeling, in which PcrV was threaded into the structure of LcrV [28] using Phyre [29] are indicated by orange lines. The fusion joint where LcrV and PcrV are fused in hybrid proteins V1 and V2 are indicated through red, vertical lines. Hybrid V3 is a replacement of the PcrV alpha helix 6 with the corresponding sequence of LcrV. The two regions exchanged in fusion V4 are indicated as well. Residue Q87, which was changed to cysteine to demonstrate crosslinking to PopD (Fig 2D) is highlighted with a red triangle.
(TIF)

S3 Fig Direct comparison of ExoS translocation by a strain producing the PopD-YopD hybrid D4 in the presence or absence of Pcr1. In order to clearly demonstrate the restoration of protein translocation by deleting *pcr1* in the context of the strain producing PopD-YopD(D4), samples from a translocation assay involving the parental (WT) strain, a strain producing the PopD-YopD(D4) hybrid, as well as Δ*pcr1* derivatives of either strain were run on the same gel. Lanes corresponding to Triton X-100 (T)- and SDS(S)-extracted samples are indicated. ExoS, G6PI, and RpoA were detected by Western blot. A schematic of the PopD-YopD hybrid D4 is depicted below the blots.
(TIF)

S4 Fig PopB forms a dimer, which can be tethered with only a minor defect in protein translocation. A) Dimer formation for PopB was assayed by infecting A549 cells with *P*. *aeruginosa* producing the indicated version of PopB (RP2349 “WT”, or RP8134 “PopB(A280C)”). Where noted, a size-tagged version of PopB (PopB-4VG) or the corresponding cysteine mutant (PopB(A280C)-4VG) were co-produced from a plasmid. Disulfide bond formation was promoted through the addition of copper-phenanthroline, where indicated. Infected cells were washed with 1M KCl to remove peripherally associated proteins and treated with iodoacetamide to block free cysteines, before membrane proteins were extracted using 0.5% Triton X-100. Protein samples were separated by SDS-PAGE and probed with an affinity purified antibody directed against PopB. The molecular weights of the two homo-dimer species, as well as that of the heterodimer consisting of PopB(A280C)and PopB(A280C)-4VG are noted. B) Translocation of strains producing PopB or PopB(A280C) was assayed through differential lysis. Extraction with Triton X-100 (T), releases proteins in the host cell cytoplasm and plasma membrane, but not bacterial proteins. SDS (S) solubilizes both bacterial and host cell proteins. The experiments were carried out in the presence of the reductant (TCEP), or the oxidant copper phenanthroline. Samples were separated by SDS-Page. ExoS, Tubulin, and RpoA were detected by Western blot. Translocation of ExoS was quantitated using ImageJ software. Translocated ExoS in cells infected with the PopB(A280C)-producing strain was normalized to translocation into cells infected with the strain producing wild-type PopB. Translocation was quantified by normalizing ExoS to G6PI in the Triton X-100 sample, which was subsequently compared to the level of translocated ExoS in the corresponding WT sample. Data are reported as the mean of four biological replicates with standard deviations. Statistical significance was calculated by unpaired t-test with Welch’s correction, comparing the PopB(A280C) sample to the corresponding wild-type. The difference in translocation in the presence of TCEP was not significant, in the presence of copper, the defect in translocation was barely significant (p = 0.03).
(TIF)

S1 Table Strains and plasmids. (PDF)

S2 Table Primers used for plasmid- and strain construction. (PDF)
