# Supplementary figures and images for: The Type III Secretion Translocation Pore Senses Host Cell Contact
Source: PLoS Pathog. 2016 Mar 29;12(3):e1005530. doi: 10.1371/journal.ppat.1005530 (PMC4811590; doi:10.1371/journal.ppat.1005530)

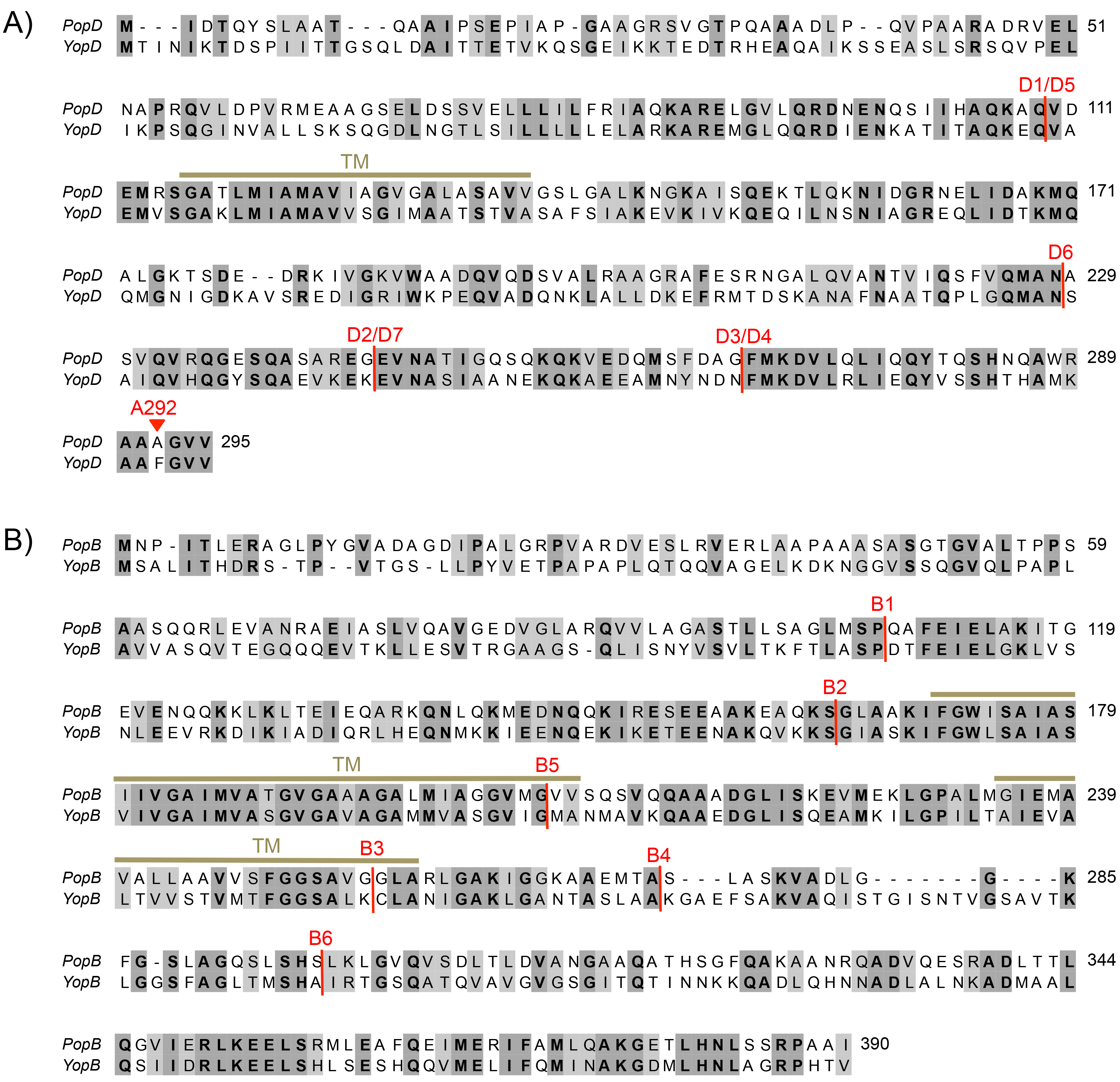

Supplement: S1 Fig — A) PopD (Genbank accession AAG05098) and YopD (Genbank accession AAA72322) were aligned using ClustalW. The amino acid sequences are 39% identical (58% similar). Identical residues are shaded dark grey, similar residues in light grey. Residue numbers next to the alignment rows correspond to the amino acid number of the P. aeruginosa translocator. Predicted transmembrane domains (TM) are indicated by olive lines above the corresponding amino-acid sequence. Residue A292 of PopD, which corresponds to F303 in YopD, is highlighted since it, when substituted with cysteine, allowed PopD to be disulfide bonded to PcrV(Q87C). Fusion junctions of hybrid proteins used in Figs 1 and 5 are highlighted in red. B) PopB (Genbank accession AAG05097) and YopB (Genbank accession AAA72321) were aligned using ClustalW. The proteins are 40% identical (65% similar). Fusion junctions of hybrid proteins in Fig 5 are highlighted in red. (TIF) [file ppat.1005530.s001.tif]

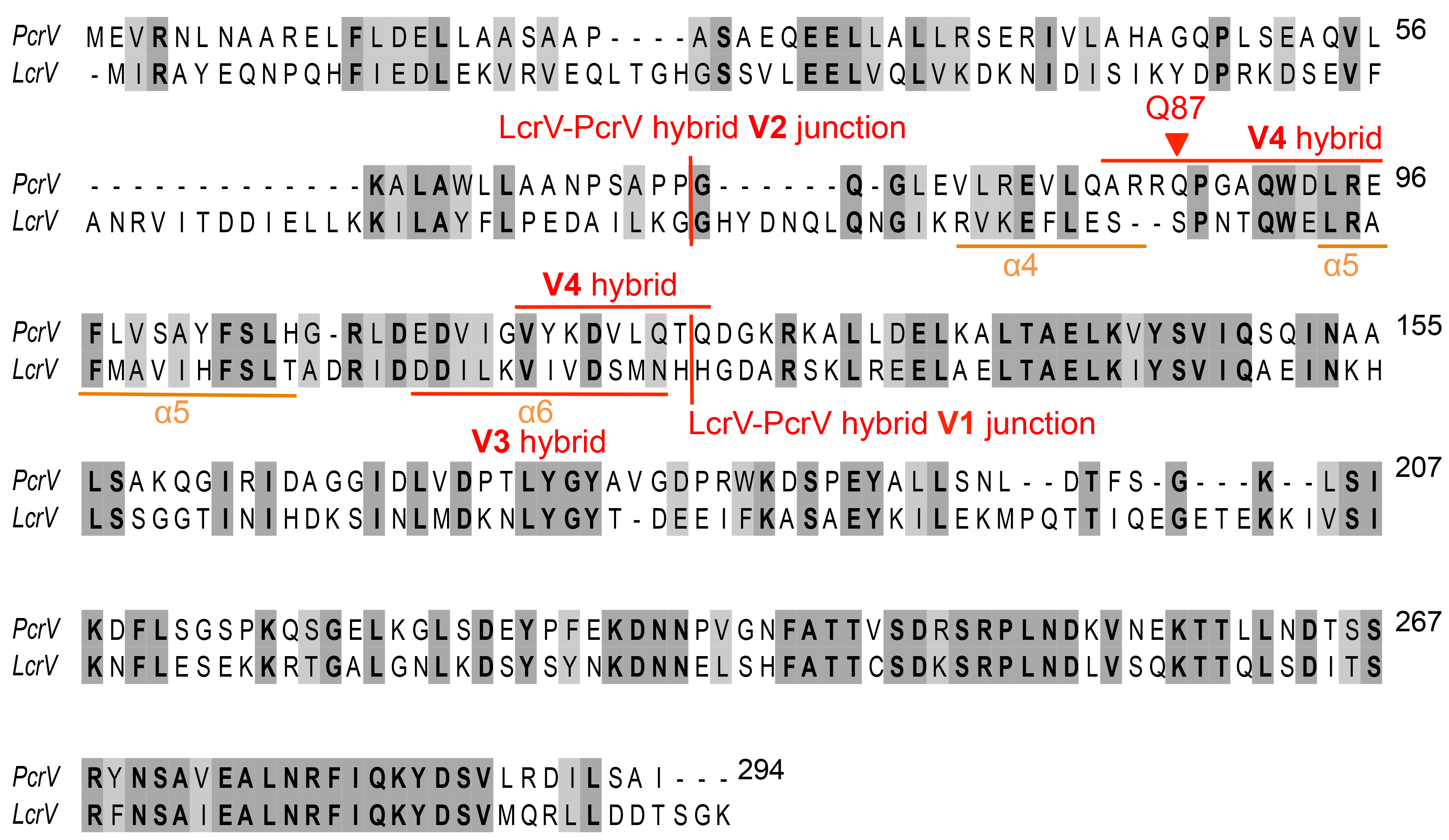

Supplement: S2 Fig — PcrV (P. aeruginosa PAO1, Genbank accession AAG05095) and LcrV (Y. pseudotuberculosis YPIII, Genbank accession AAA27645) were aligned using ClustalW in MacVector (MacVector Inc.). Identical residues are shaded dark grey, similar amino acids are shaded light grey. Overall, PcrV and LcrV are 37% identical (54% similar). α- helices 4, 5, and 6, based on structural modeling, in which PcrV was threaded into the structure of LcrV [28] using Phyre [29] are indicated by orange lines. The fusion joint where LcrV and PcrV are fused in hybrid proteins V1 and V2 are indicated through red, vertical lines. Hybrid V3 is a replacement of the PcrV alpha helix 6 with the corresponding sequence of LcrV. The two regions exchanged in fusion V4 are indicated as well. Residue Q87, which was changed to cysteine to demonstrate crosslinking to PopD (Fig 2D) is highlighted with a red triangle. (TIF) [file ppat.1005530.s002.tif]

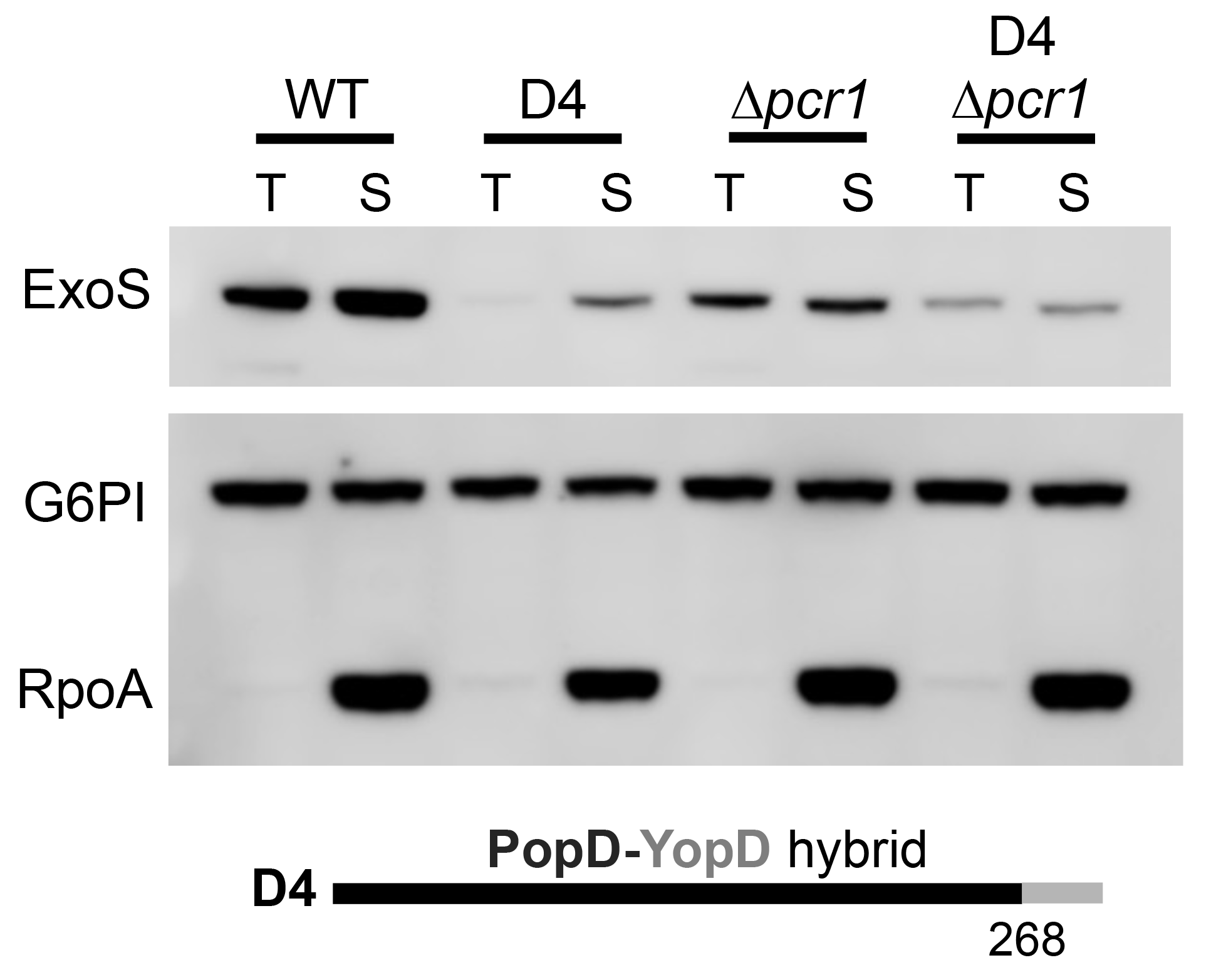

Supplement: S3 Fig — In order to clearly demonstrate the restoration of protein translocation by deleting pcr1 in the context of the strain producing PopD-YopD(D4), samples from a translocation assay involving the parental (WT) strain, a strain producing the PopD-YopD(D4) hybrid, as well as Δpcr1 derivatives of either strain were run on the same gel. Lanes corresponding to Triton X-100 (T)- and SDS(S)-extracted samples are indicated. ExoS, G6PI, and RpoA were detected by Western blot. A schematic of the PopD-YopD hybrid D4 is depicted below the blots. (TIF) [file ppat.1005530.s003.tif]

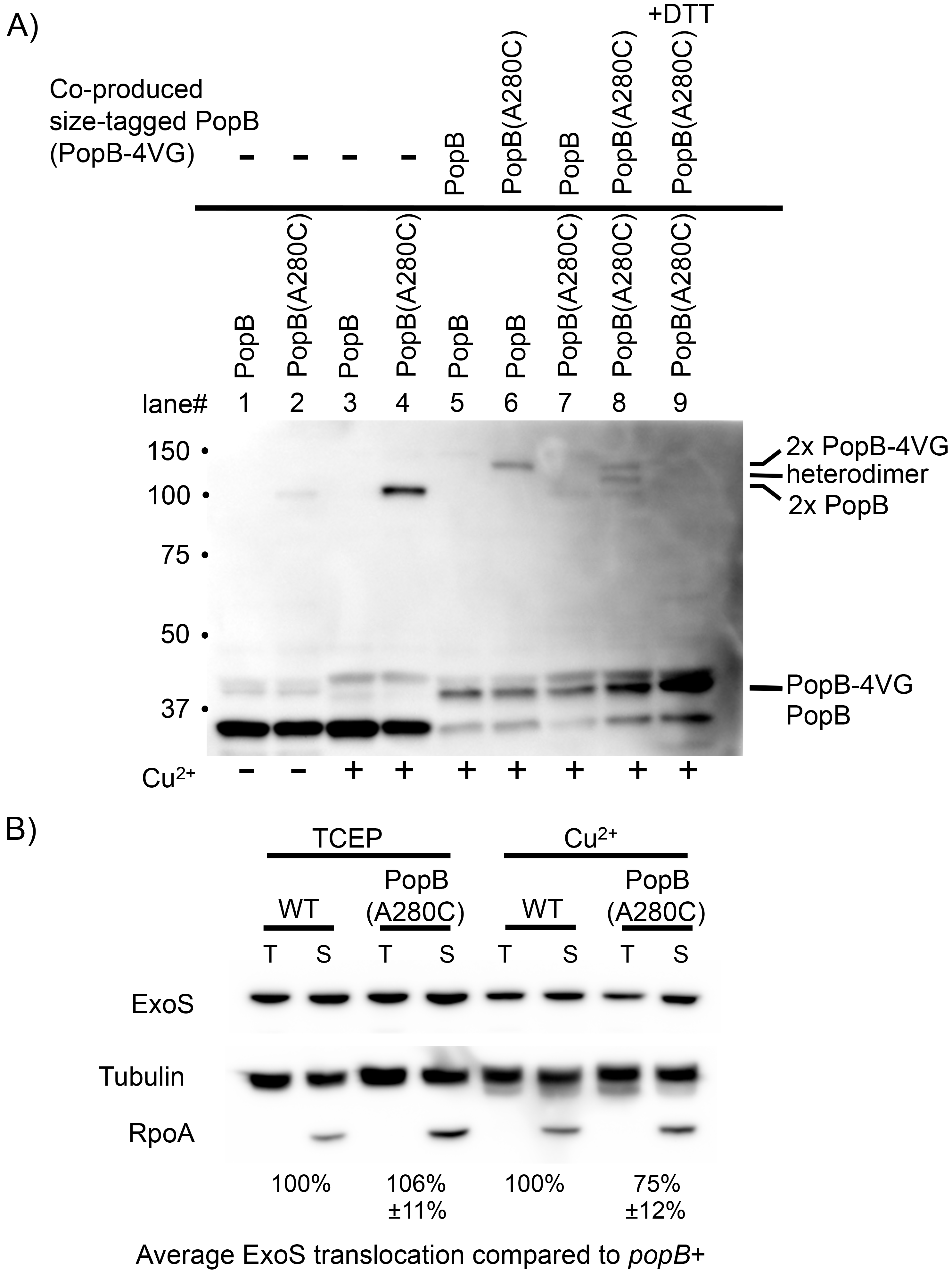

Supplement: S4 Fig — A) Dimer formation for PopB was assayed by infecting A549 cells with P. aeruginosa producing the indicated version of PopB (RP2349 “WT”, or RP8134 “PopB(A280C)”). Where noted, a size-tagged version of PopB (PopB-4VG) or the corresponding cysteine mutant (PopB(A280C)-4VG) were co-produced from a plasmid. Disulfide bond formation was promoted through the addition of copper-phenanthroline, where indicated. Infected cells were washed with 1M KCl to remove peripherally associated proteins and treated with iodoacetamide to block free cysteines, before membrane proteins were extracted using 0.5% Triton X-100. Protein samples were separated by SDS-PAGE and probed with an affinity purified antibody directed against PopB. The molecular weights of the two homo-dimer species, as well as that of the heterodimer consisting of PopB(A280C)and PopB(A280C)-4VG are noted. B) Translocation of strains producing PopB or PopB(A280C) was assayed through differential lysis. Extraction with Triton X-100 (T), releases proteins in the host cell cytoplasm and plasma membrane, but not bacterial proteins. SDS (S) solubilizes both bacterial and host cell proteins. The experiments were carried out in the presence of the reductant (TCEP), or the oxidant copper phenanthroline. Samples were separated by SDS-Page. ExoS, Tubulin, and RpoA were detected by Western blot. Translocation of ExoS was quantitated using ImageJ software. Translocated ExoS in cells infected with the PopB(A280C)-producing strain was normalized to translocation into cells infected with the strain producing wild-type PopB. Translocation was quantified by normalizing ExoS to G6PI in the Triton X-100 sample, which was subsequently compared to the level of translocated ExoS in the corresponding WT sample. Data are reported as the mean of four biological replicates with standard deviations. Statistical significance was calculated by unpaired t-test with Welch’s correction, comparing the PopB(A280C) sample to the corresponding wild-type. [file ppat.1005530.s004.tif]
