# Supplementary material for: The Type III Secretion Translocation Pore Senses Host Cell Contact
Source: PLoS Pathog. 2016 Mar 29;12(3):e1005530. doi: 10.1371/journal.ppat.1005530 (PMC4811590; doi:10.1371/journal.ppat.1005530)
Supplement: S1 Table — (PDF) [file ppat.1005530.s005.pdf]

Table S1. Strains and plasmids

Strain #	Genotype	hybrid translocator	Reference
RP2318	PAO1F *ΔexsE ΔexoT ΔexoY*		[1]
RP2349	PAO1F *ΔexsE ΔexoT ΔexoY exoS(GAP-/ADPR-)*		[1]
RP2888	PAO1F *ΔexsE ΔexoT ΔexoY exoS(GAP-/ADPR-) ΔpopD*		This study
RP2996	PAO1F *ΔexsE ΔexoT ΔexoY Δpcr1*		This study
RP3624	PAO1F *ΔexsE ΔexoT ΔexoY ΔpcrHpopBD*		This study
RP3714	PAO1F *ΔexsE ΔexoT ΔexoY ΔpcrHpopBD lcrV(N)-pcrV(C)*	**V1**	This study
RP6592	PAO1F *ΔexsE ΔexoT ΔexoY ΔpcrHpopBD lpv72*	**V2**	This study
RP5891	PAO1F *ΔexsE ΔexoT ΔexoY ΔpcrHpopBD pcrV-α6-lcrV*	**V3**	This study
RP6225	PAO1F *ΔexsE ΔexoT ΔexoY ΔpcrHpopBD pcrV-α456-lcrV*	**V4**	This study
RP3670	PAO1F *ΔexsE ΔexoT ΔexoY exoS(GAP-/ADPR-) ΔpcrHpopBD*		This study
RP6369	PAO1F *∆exsE ∆exoT ∆exoY ∆pcr1* "S+" *∆pcrHpopBD*		This study
RP6370	PAO1F *ΔexsE ΔexoT ΔexoY exoS(GAP-/ADPR-) ΔpcrHpopBD Δpcr1*		This study
RP6641	PAO1F *ΔexsE ΔexoT ΔexoY exoS(GAP-/ADPR-) pcrV(Q87C) ΔpopD*		This study
RP8134	PAO1F *ΔexsE ΔexoT ΔexoY exoS(GAP-/ADPR-) popB(A280C)*		This study
RP8136	PAO1F *ΔexsE ΔexoT ΔexoY exoS(GAP-/ADPR-) popD(R243C)*		This study
RP8766	PAO1F *ΔexsE ΔexoT ΔexoY exoS(GAP-/ADPR-) Δpcr1 popD(R243C)*		This study
RP9266	PAO1F *ΔexsE ΔexoT ΔexoY exoS(GAP-/ADPR-) Δpcr1*		This study
RP9268	PAO1F *ΔexsE ΔexoT ΔexoY exoS(GAP-/ADPR-) ΔpcrV2*		This study
RP9270	PAO1F *ΔexsE ΔexoT ΔexoY exoS(GAP-/ADPR-) PYD268*		This study
RP9272	PAO1F *ΔexsE ΔexoT ΔexoY exoS(GAP-/ADPR-) lcrV(N)-pcrV(C)*		This study
RP9274	PAO1F *ΔexsE ΔexoT ΔexoY exoS(GAP-/ADPR-) lcrV(N)-pcrV(C) PYD268*		This study
RP9276	PAO1F *ΔexsE ΔexoT ΔexoY exoS(GAP-/ADPR-) Δpcr1 PYD268*	**D4**	This study

Plasmids

Plasmid	Relevant features		Reference
pPSV37	colE1 origin, gentR, PA origin, oriT, lacUV5 promoter, lacIq, stops in every reading frame preceding the MCS and T7 terminator following the MCS (relative to the lacUV5 promoter)		[2]
pEXG2	allelic exchange vector, colE1 origin, oriT, gentamicin resistance, sacB		[3]
pEXG2-Δ*pcrV2*	delete codons 4-180 of ∆*pcrV2*		[1]
pEXG2-Δ*pcr1*	delete codons 10–81 of *pcr1*		[4]
pEXG2-*pcrV(Q87C)*	introduce Q87C mutation into *pcrV*		This study
pEXG2-*popB(A280C)*	introduce A280C mutation into *popB*		This study
pEXG2-*popD(R243C)*	introduce R243C mutation into *popD*		This study
pEXG2-*lcrV(N)-pcrV(C)*	replace *pcrV* with *lcrV(1-145)-pcrV(124-294)* hybrid	**V1**	This study
pEXG2-*LPV72*	replace *pcrV* with *lcrV(1-G87)-pcrV(G72-294)* hybrid	**V2**	This study
pEXG2-*pcrV-α6-lcrV*	replace *pcrV* with *pcrV*(1-110)-*lcrV*(133-144)-*pcrV*(123-294) fusion replacing α6	**V3**	This study
pEXG2-*pcrV-α456-lcrV*	*pcrV* with codons A84-E96 replaced by *lcrV* codons L105-A117, and pcrV V116-Q124 replaced by lcrV V138-H146 (portions of α4, α5, and α6)	**V4**	
pEXG2-*PYD268*	replace *popD* with *popD(1-268)-yopD(280-306)* hybrid	**D4**	This study
pP37-*lcrHpcrH*	translationally coupled *lcrH* and *pcrH* genes		This study
pP37-*lcrHpcrH-popBD*	plasmid encoding wild type *popB* and *popD* under control of the *lacUV5* promoter in pPSV37		This study
pP37-*lcrHpcrH-popByopD*	plasmid encoding wild type *popB* and *yopD* under control of the *lacUV5* promoter in pPSV37		This study
pP37-*lcrHpcrH-yopBpopD*	plasmid encoding wild type *yopB* and *popD* under control of the *lacUV5* promoter in pPSV37		This study
pP37-*lcrHpcrH-yopBD*	plasmid encoding wild type *yopB* and *yopD* under control of the *lacUV5* promoter in pPSV37		This study
pP37-*lcrHpcrH-popBYPD110*	plasmid encoding *popB* and a *yopD(1-118)-popD(110-295)* fusion under control of the lacUV5 promoter in pPSV37	**D1**	This study

pP37-*popD*	Plasmid encoding wild type PopD under control of the lacUV5 promoter in pPSV37		[4]
pP37-*popD*(A292C)	plasmid encoding *popD* with codon Ala292 changed to Cys under control of the lacUV5 promoter in pPSV37		This study
pP37-*lcrHpcrH-popBYPD246*	plasmid encoding *popB* and a *yopD(1-256)-popD(246-295)* fusion under control of the lacUV5 promoter in pPSV37	**D2**	This study
pP37-*lcrHpcrH-popBYPD269*	plasmid encoding *popB* and a *yopD(1-279)-popD(269-295)* fusion under control of the lacUV5 promoter in pPSV37	**D3**	This study
pP37-*lcrHpcrH-popByopD(F303A)*	plasmid encoding *popB* and *yopD(F330A)* under control of the lacUV5 promoter in pPSV37		This study
pP37-*lcrHpcrH-popByopD(R268Q)*	plasmid encoding *popB* and *yopD(R268Q)* under control of the lacUV5 promoter in pPSV37		This study
pP37-*lcrHpcrH-popByopD(E289Q)*	plasmid encoding *popB* and *yopD(E289Q)* under control of the lacUV5 promoter in pPSV37		This study
pP37-*lcrHpcrH-popByopD(V292T)*	plasmid encoding *popB* and *yopD(V292T)* under control of the lacUV5 promoter in pPSV37		This study
pP37-*lcrHpcrH-popByopD(S293Q)*	plasmid encoding *popB* and *yopD(S293Q)* under control of the lacUV5 promoter in pPSV37		This study
pP37-*lcrHpcrH-popByopD(T296N)*	plasmid encoding *popB* and *yopD(T296N)* under control of the lacUV5 promoter in pPSV37		This study
pP37-*lcrHpcrH-popByopD(H297Q)*	plasmid encoding *popB* and *yopD(H297Q)* under control of the lacUV5 promoter in pPSV37		This study
pP37-*lcrHpcrH-popByopD(M299W)*	plasmid encoding *popB* and *yopD(M299W)* under control of the lacUV5 promoter in pPSV37		This study
pP37-lcrHpcrH-popByopD(K300R)	plasmid encoding *popB* and *yopD(K300R)* under control of the lacUV5 promoter in pPSV37		This study
pP37-*lcrHpcrH-popB(A280C)popD*	plasmid encoding *popB(A280C)* and *popD* under control of the lacUV5 promoter in pPSV37		This study
pP37-popD172-4V	plasmid encoding *popD* into which four copies of the VSV-G epitope tag were inserted after codon 172 under control of the *lacUV5* promoter in pPSV37		This study
pP37-popD(R243C)172-4V	plasmid encoding *popD(R243C)* into which four copies of the VSV-G epitope tag were inserted after codon 172 under control of the *lacUV5* promoter in pPSV37		This study
pP37-popB225-4V	plasmid encoding *popB* into which four copies of the VSV-G epitope tag were inserted after codon 225 under control of the *lacUV5* promoter in pPSV37		This study
pP37-popB(A280C)225-4V	plasmid encoding *popB(A280C)* into which four copies of the VSV-G epitope tag were inserted after codon 225 under control of the *lacUV5* promoter in pPSV37		This study
pP37-*lcrHpcrH-popBpopD(R243C)*	plasmid encoding *popB* and *popD(R243C)* under control of the lacUV5 promoter in pPSV37		This study
pP37-*lcrHpcrH-yopBYPD110*	plasmid encoding *yopB* and a *yopD(1-118)-popD(110-295)* fusion under control of the lacUV5 promoter in pPSV37		This study
pP37-*lcrHpcrH-yopBYPD246*	plasmid encoding *yopB* and a *yopD(1-256)-popD(246-295)* fusion under control of the lacUV5 promoter in pPSV37		This study
pP37-*lcrHpcrH-yopBYPD269*	plasmid encoding *yopB* and a *yopD(1-279)-popD(269-295)* fusion under control of the lacUV5 promoter in pPSV37		This study
pP37-*lcrHpcrH-yopBPYD109*	plasmid encoding *yopB* and a *popD(1-109)-yopD(119-306)* fusion under control of the lacUV5 promoter in pPSV37		This study
pP37-*lcrHpcrH-yopBPYD228*	plasmid encoding *yopB* and a *popD(1-228)-yopD(240-306)* fusion under control of the lacUV5 promoter in pPSV37		This study
pP37-*lcrHpcrH-yopBPYD245*	plasmid encoding *yopB* and a *popD(1-245)-yopD(257-306)* fusion under control of the lacUV5 promoter in pPSV37		This study
pP37-*lcrHpcrH-YPB108popD*	plasmid encoding a *yopB(1-103)-popB(108-390)* hybrid and *popD* under control of the lacUV5 promoter in pPSV37		This study
pP37-*lcrHpcrH-YPB165popD*	plasmid encoding a *yopB(1-160)-popB(165-390)* hybrid and *popD* under control of the lacUV5 promoter in pPSV37		This study
pP37-*lcrHpcrH-YPB255popD*	plasmid encoding a *yopB(1-251)-popB(255-390)* hybrid and *popD* under control of the lacUV5 promoter in pPSV37		This study
pP37-*lcrHpcrH-YPB274popD*	plasmid encoding a *yopB(1-269)-popB(274-390)* hybrid and *popD* under control of the lacUV5 promoter in pPSV37		This study
pP37-*lcrHpcrH-PYB206popD*	plasmid encoding a *popB(1-206)-yopB(203-390)* hybrid and *popD* under control of the lacUV5 promoter in pPSV37		This study
pP37-*lcrHpcrH-PYB297popD*	plasmid encoding a *popB(1-297)-yopB(309-390)* hybrid and *popD* under control of the lacUV5 promoter in pPSV37		This study

Literature Cited

1. Cisz M, Lee PC, Rietsch A. ExoS controls the cell contact-mediated switch to effector secretion in Pseudomonas aeruginosa. J Bacteriol. 2008;190(8):2726-38. Epub 2007/11/28. doi: JB.01553-07 [pii] 10.1128/JB.01553-07. PubMed PMID: 18039770; PubMed Central PMCID: PMCPMC2293250.
2. Lee PC, Stopford CM, Svenson AG, Rietsch A. Control of effector export by the Pseudomonas aeruginosa type III secretion proteins PcrG and PcrV. Mol Microbiol. 2010;75(4):924-41. Epub 2010/05/22. doi: 10.1111/j.1365-2958.2009.07027.x. PubMed PMID: 20487288; PubMed Central PMCID: PMC3124366.
3. Rietsch A, Vallet-Gely I, Dove SL, Mekalanos JJ. ExsE, a secreted regulator of type III secretion genes in Pseudomonas aeruginosa. Proc Natl Acad Sci U S A. 2005;102(22):8006-11. Epub 2005/05/25. doi: 10.1073/pnas.0503005102. PubMed PMID: 15911752; PubMed Central PMCID: PMC1142391.
4. Tomalka AG, Stopford CM, Lee PC, Rietsch A. A translocator-specific export signal establishes the translocator-effector secretion hierarchy that is important for type III secretion system function. Mol Microbiol. 2012;86(6):1464-81. Epub 2012/11/06. doi: 10.1111/mmi.12069. PubMed PMID: 23121689; PubMed Central PMCID: PMC3524397.
