# Supplementary material for: The Type III Secretion Translocation Pore Senses Host Cell Contact
Source: PLoS Pathog. 2016 Mar 29;12(3):e1005530. doi: 10.1371/journal.ppat.1005530 (PMC4811590; doi:10.1371/journal.ppat.1005530)
Supplement: S2 Table — (PDF) [file ppat.1005530.s006.pdf]

Table S2. Primers used for plasmid- and strain construction

Primer name	Sequence	Description
pcrVQ87C-3-1	CTCCGCGAAGTCCTGCAGGCACGTCGGtgtCCCGGTGCGCA GTGGGATCTG	pair with pcrVEX3 to amplify 3' flank, changing Q87 to C in *pcrV*
pcrVEX3	AAAAAaagcttGGATACCTCTAGATCGCGCTGAGAA	*pcrV* 3' primer with HindIII
pcrV5-1	AAAAAgaattcCGAGCCCGCCGACGGCTGGTTCGA	5' flank primer with EcoRI site
pcrVQ87C-5-2	CAGATCCCACTGCGCACCGGGACACCGACGTGCCTGCAGG ACTTCGCGGAG	pair with pcrV5-1 to amplify 5' flank, changing Q87 to C in *pcrV*
pcrVQ87Ctest	GTCCTGCAGGCACGTCGGtgt	test primer to confirm mutation has been crossed onto the chromosome
popB5R2	AAAAAGAATTCGAAACATCAGGAGAAGGCAACCATCATGAA TCCGATAACGCTTGAACGC	5' primer to amplify *popB* with EcoRI site
BA280C-5-2	CAAACTGCCGAACTTGCCGCCCAGGTCGCAGACCTTGCTC GCCAGGCTGGCGGT	pair with popB5R2 to amplify 5' flank, changing A280 to C in *popB*
BA280C-3-1	ACCGCCAGCCTGGCGAGCAAGGTCtgcGACCTGGGCGGCA AGTTCGGCAGTTTG	pair with popBEX3 to amplify 3' flank, changing A280 to C in *popB*
popBEX3	AAAAAaagcttGACGTCTCCTCAGATCGCTGCCGGT	3' primer to amplify popB with HindIII primer
BA280Ctest	CGCCAGCCTGGCGAGCAAGGTCTG	test primer to confirm mutation has been crossed onto the chromosome
popD5R	AAAAAgaattcTTAGGAGGCGCCCCCATGATCGACACGCAATA TTCCCT	5' primer for *popD* ORF with EcoRI site
DR243C-5-2	GATGGTGGCGTTGACCTCTCCTTCGCAGGCGCTCGCCTGC GATTCGCCCTGGCGCAC	pair with popD5R to amplify 3' flank, changing R243 to C in *popD*
DR243C-3-1	GTGCGCCAGGGCGAATCGCAGGCGAGCGCCtgcGAAGGAG AGGTCAACGCCACCATC	pair with popDEX3 (for pPSV37) or popD3-2 (for pEXG2) to amplify 3' flank, changing R243 to C in *popD*
popDEX3	AAAAAaagcttCGCGCGGAGACGGCTCAGACCACT	*popD* 3' primer with HindIII
popD3-2	AAAAAaagcttAGGGTCAGTTGCGCTGCGAGAAT	*popD* 3' flank primer with HindIII
DR243Ctest	GGCGAATCGCAGGCGAGCGCCTGC	test primer to confirm mutation has been crossed onto the chromosome
LcrVNfP-5-2	CCTTGCGCTTGCCGTCCTGATGATTCATTGAATCAACAATC ACT	LcrV N-term - PcrV C-term fusion, x-over, pair with LcrVNfUS-5-1 to amplify *lcrV* portion
LcrVNfP-3-1	AGTGATTGTTGATTCAATGAATCATCAGGACGGCAAGCGCA AGG	LcrV N-term - PcrV C-term fusion, x-over, pair with pcrVEX3 to amplify 3' *pcrV* portion
LcrVNfUS-5-1	TGTTGATCTGAGGAATCACGATGATTAGAGCCTACGAACAA AACC	LcrV N-term - PcrV C-term fusion, x-over, pair with LcrVNfP-5-2 to amplify *lcrV* portion
LPV87-3-1	CTACCCGAGGATGCCATTCTTAAAGGCGGGCAGGGCCTCG AGGTACTCCGCGAAG	LPV72!, LcrV(1-G87)-PcrV(G72-end), pair with LcrVNfUS-5-1
LPV87-5-2	CTTCGCGGAGTACCTCGAGGCCCTGCCCGCCTTTAAGAAT GGCATCCTCGGGTAG	LPV72!, LcrV(1-G87)-PcrV(G72-end), pair with pcrVEX3 to amplify 3' *pcrV* portion
PLPa5-3-1	CATTGAATCAACAATCACTTTCAAAATATCATCGTCGAGACG CCCGTGCAGGCT	*pcrV*(1-110)-*lcrV*(133-144)-*pcrV*(123-end) fusion replacing α6 (not α 5, model was revised), 3' flank primer
PLPa5-5-2	GATATTTTGAAAGTGATTGTTGATTCAATGAATACCCAGGAC GGCAAGCGCAAG	*pcrV*(1-110)-*lcrV*(133-144)-*pcrV*(123-end) fusion replacing α6 (not α 5, model was revised), 5' flank primer
PLP345-3-1	GTGTCGGCCTATTTCAGCCTGCACGGGCGTCTCGACGAGG ATGTCATCGGTGTGATTGTTGATTCAATGAATCATCATGACG GCAAGCGCAAGGCGCTG	*pcrV* with codons A84-E96 replaced by *lcrV* codons L105-A117, and pcrV V116-Q124 replaced by lcrV V138-H146 (portions of α4, α5, and α6) – primer for 3' flank
PLP345-5-2	CGCCCGTGCAGGCTGAAATAGGCCGACACCAGGAACGCCC GCAATTCCCATTGTGTATTCGGCGATGATTCAAGCTGCAGG ACTTCGCGGAGTAC	*pcrV* with codons A84-E96 replaced by *lcrV* codons L105-A117, and pcrV V116-Q124 replaced by lcrV V138-H146 (portions of α4, α5, and α6) – primer for 5' flank
pcrVUSfl-5-2	GGTTTTGTTCGTAGGCTCTAATCATCGTGATTCCTCAGATCA ACA	pair with pcrV5-1 to amplify upstream sequences of *pcrV* and fuse to *lcrV*
PYD268-5-2	CCTTTTCCTCCCGCGCGGAGACGGCTCAGACAACACCAAA AGCGGCTTTC	pair with popD5R to amplify PYD268 5' flank to insert into chromosome

PYD268-3-1	GAAAGCCGCTTTTGGTGTTGTCTGAGCCGTCTCCGCGCGGGAGGAAAAGG	pair with popD3-2 to amplify PYD268 3' flank to insert into chromosome
lcrH5R	AAAAAgaattcTTGAGGAGGCGCCCCCATGCAACAAGAGACGACAG	5' lcrH primer with EcoRI site and stop in alpha fragment
lcrHfp	TCGGAAGGGGTCGGCTGGTTCATGGGTTATCAACGCACTC	lcrH 3' primer to fuse with 5' end of pcrH
pcrHfl	GAGTGCGTTGATAACCCATGAACCAGCCGACCCCTTCCGA	pcrH 5' primer to fuse with 3' end of lcrH
pcrH3K	TATATggtaccTCAAGCGTTATCGGATTCATAT	pcrH 3' primer with KpnI site
yopB5Kpn	TATATggtaccGTTTAAGGAGGAATAACCATGAGTGCGTTGATAACC	yopB 5' primer with KpnI site
yopB3Bam	AAAAAggatccCTAAACAGTATGGGGTCTGCCG	yopB 3' primer with BamHI site
popB5Kpn	TATATggtaccGTTTAAGGAGGAATAACCATGAATCCGATAACGCTTGAA	popB 5' primer with KpnI site
popB3Bam	AAAAAggatccTCAGATCGCTGCCGGTCGGCTGGA	popB 3' primer with BamHI site
yopD5Bam	TATATggatccGTTTAAGGAGGAATAACCATGACAATAAATATCAAG	yopD 5' primer with BamHI site
yopD3Sal	AAAAAgtcgacTCAGACAACACCAAAAGCGGC	yopD 3' primer with SalI site
popD5Bam	TATATggatccGTTTAAGGAGGAATAACCATGATCGACACGCAATATTCCCT	popD 5' primer with BamHII site
popD3Sal	AAAAAgtcgacCGCGCGGAGACGGCTCAGACCACT	popD 3' primer with SalI site
popDTM3-5	CTACTATTACTGCCCAAAAGGAGCAGGTGGACGAGATGCGCAGCGG	popD 5' primer for 3' half w/ TM (YPD110)
yopD5-3	CGCTGCGCATCTCGTCCACCTGCTCCTTTTGGGCAGTAATAGTAG	yopD 3' primer for 5' half w/o TM (YPD110)
PopDA292C-3H	AAAAAAAGCTTTCAGACCACTCCGCACGCCGCACGCCAGGCCTGGTTATG	mutate cdon 292 of *popD* to Cys, ORF primer with HindIII site
YPD246-5	CTCAAGCCGAGGTCAAAGAGAAAGAGGTCAACGCCACCATCGGGCAG	fuse PopD E246-295 to YopD 1-K256, 5' primer for *popD* piece
YPD246-3	CTGCCCGATGGTGGCGTTGACCTCTTTCTCTTTGACCTCGGCTTGAG	3' primer for *yopD* piece
YPD269-5	GCGATGAACTATAATGATAACTTCATGAAGGACGTCCTGCAG	fuse PopD F269-295 to YopD 1-N279, 5' primer for *popD* piece
YPD269-3	CTGCAGGACGTCCTTCATGAAGTTATCATTATAGTTCATCGC	3' primer for *yopD* piece
yopDF303A-3Sal	AAAAAgtcgacTCAGACAACACCGGCAGCGGCTTTCATGGCGTGAGTATGACTG	*yopD* 3'end point mutations that change to corresponding popD codon, with SalI site
yopDV292T-3Sal	AAAAAgtcgacTCAGACAACACCAAAAGCGGCTTTCATGGCGTGAGTATGACTGCTGGTATATTGTTCAATCAAGCG	
yopDS293Q-3Sal	AAAAAgtcgacTCAGACAACACCAAAAGCGGCTTTCATGGCGTGAGTATGACTCTGAACATATTGTTCAATCAAGCG	
yopDT296N-3Sal	AAAAAgtcgacTCAGACAACACCAAAAGCGGCTTTCATGGCGTGGTTATGACTGCTAACATATTGTTCAATCAAGCG	
yopDH297Q-3Sal	AAAAAgtcgacTCAGACAACACCAAAAGCGGCTTTCATGGCCTGAGTATGACTGCTAACATATTGTTCAATCAAGCG	
yopDM299W-3Sal	AAAAAgtcgacTCAGACAACACCAAAAGCGGCTTTCCAGGCGTGAGTATGACTGCTAACATATTGTTCAATC	
yopDK300R-3Sal	AAAAAgtcgacTCAGACAACACCAAAAGCGGCACGCATGGCGTGAGTATGACTGCTAACATATTG	
D172-4V-5-2	TCGATATCGGTGTACTTGCCCAGCCGGTTCATCTCGATGTCGGTGTATTTTCCTAATCTATTCATTTCAATATCTGTATACGCCTGCATCTTCGCGTCGA	amplify 4x VSV-G tag and insert after codon 172 of *popD* , pair with popD5R
D172-4V-3-1	CGGCTGGGCAAGTACACCGATATCGAAATGAACAGGCTCGGGAAGTACACCGACATTGAAATGAATCGCCTAGGAAAGCTTGGCAAGACCTCCGACGAG	amplify 4x VSV-G tag and insert after codon 172 of *popD*, pair with popDEX3
B2254V-3-1	GGCTGGGCAAGTACACCGATATCGAAATGAACAGGCTCGGGAAGTACACCGACATTGAAATGAATCGCCTAGGAAAGATGGAAAAGCTCGGCCCGGCACT	insert 4xVSVG after codon 225 of *popB*, pair with popB5R2
B2254V-5-2	CGATATCGGTGTACTTGCCCAGCCGGTTCATCTCGATGTCGGTGTATTTTCCTAATCTATTCATTTCAATATCTGTATACACTTCCTTGCTGATCAATCC	insert 4xVSVG after codon 225 of *popB*, pair with popBEX3
popD5-3	GCTGACCATCTCCGCTACCTGCGCCTTCTGCGCGTGGATGATC	*popD* 3' primer for 5' half of gene, w/o TM (PYD109)
yopTM3-5	GATCATCCACGCGCAGAAGGCGCAGGTAGCGGAGATGGTCAGC	*yopD* 3' half and TM primer for fusion to popD 5' arm(PYD109)
PYD228-3-1	CAGTCCTTCGTCCAGATGGCCAACAGTGCGATTCAAGTTCATCAAGGG	PopD 1-N228 fused to YopD S240-end, pair with yopD3Sal

PYD228-5-2	CCCTTGATGAACTTGAATCGCACTGTTGGCCATCTGGACGA AGGACTG	PopD 1-N228 fused to YopD S240-end, pair with popD5Bam
PYD245-3-1	TCGCAGGCGAGCGCCCGGGAAGGAGAAGTCAATGCAAGTA TTGCTGCC	PopD(1-245)-YopD(257-306), pair with yopD3Sal
PYD245-5-2	GGCAGCAATACTTGCATTGACTTCTCCTTCCCGGGCGCTCG CCTGCGA	PopD(1-245)-YopD(257-306), pair with popD5Bam
YPB103-3-1	ACGAAGTTTACGCTTGCTTCACCTCAGGCGTTCGAGATCGA GCTGGCG	YopB (1-P103)-PopB(Q108-end), note YPB108!, pair with popB3Bam
YPB103-5-2	CGCCAGCTCGATCTCGAACGCCTGAGGTGAAGCAAGCGTA AACTTCGT	YopB (1-P103)-PopB(Q108-end), note YPB108!, pair with yopB5Kpn
YPB165-3-1	GCCAAGCAAGTCAAGAAATCCGGTCTGGCAGCCAAAATCTT TGGTTGG	YopB(1-160)-PopB(165-390), pair with popB3Bam
YPB165-5-2	CCAACCAAAGATTTTGGCTGCCAGACCGGATTTCTTGACTT GCTTGGC	YopB(1-160)-PopB(165-390), pair with yopB5Kpn
YPB255-3-1	CCTTTGGCGGTTCGGCACTAGGCGGGCTGGCCAGGCTGG GCG	YopB(1-250)-PopB(255-end), pair with popB3Bam
YPB255-5-2	CGCCCAGCCTGGCCAGCCCGCCTAGTGCCGAACCGCCAAA GG	YopB(1-250)-PopB(255-end), pair with yopB5Kpn
YPB274-3-1	GCTAACACCGCAAGTCTTGCGGCTAGCCTGGCGAGCAAGG TCGCCGAC	YopB(1-A269)-PopB(S274-end), pair with popB3Bam
YPB274-5-2	GTCGGCGACCTTGCTCGCCAGGCTAGCCGCAAGACTTGCG GTGTTAGC	YopB(1-A269)-PopB(S274-end), pair with yopB5Kpn
PYB206-3-1	GATGATCGCGGGCGGTGTCATGGGAATGGCGAATATGGCA GTGAAACAAGCG	PopB(1-G206)-YopB(M203-end), pair with yopB3Bam
PYB206-5-2	CGCTTGTTTCACTGCCATATTCGCCATTCCCATGACACCGC CCGCGATCATC	PopB(1-G206)-YopB(M203-end), pair with popB5Kpn
PYB297-3-1	GCCAATCGCTGTCGCACTCGATCCGTACAGGATCACAGGC AACA	PopB(1-S297)-YopB(I309-401), pair with yopB3Bam
PYB297-5-2	TGTTGCCTGTGATCCTGTACGGATCGAGTGCGACAGCGATT GGC	PopB(1-S297)-YopB(I309-401), pair with popB5Kpn
